# Integrating Physiology, Transcriptome, and Metabolome Analyses Reveals the Drought Response in Two Quinoa Cultivars with Contrasting Drought Tolerance

**DOI:** 10.3390/ijms252212188

**Published:** 2024-11-13

**Authors:** Yang Wang, Yang Wu, Qinghan Bao, Huimin Shi, Yongping Zhang

**Affiliations:** 1 College of Agriculture, Inner Mongolia Agricultural University, Hohhot 010018, China; wangyang368@jlnu.edu.cn (Y.W.); wuyang@imau.edu.cn (Y.W.); baoqinghan@jlnu.edu.cn (Q.B.); 2 College of Life Sciences, Jilin Normal University, Siping 136000, China

**Keywords:** quinoa, drought, multi-omics, Chla/Chlb, α-linolenic acid metabolism, methyl jasmonate

## Abstract

Quinoa (*Chenopodium quinoa* Willd.) is an annual broadleaf plant belonging to the Amaranthaceae family. It is a nutritious food crop and is considered to be drought-tolerant, but drought is still one of the most important abiotic stress factors limiting its yield. Quinoa responses to drought are related to drought intensity and genotype. This study used two different drought-responsive quinoa cultivars, LL1 (drought-tolerant) and ZK1 (drought-sensitive), to reveal the important mechanisms of drought response in quinoa by combining physiological, transcriptomic, and metabolomic analyses. The physiological analysis indicated that Chla/Chlb might be important for drought tolerance in quinoa. A total of 1756 and 764 differentially expressed genes (DEGs) were identified in LL1 and ZK1, respectively. GO (Gene Ontology) enrichment analysis identified 52 common GO terms, but response to abscisic acid (GO:0009737) and response to osmotic stress (GO:0006970) were only enriched in LL1. KEGG (Kyoto Encyclopedia of Genes and Genomes) analysis revealed that glycerophospholipid metabolism (ko00564) and cysteine and methionine metabolism (ko00270) ranked at the top of the list in both cultivars. A total of 1844 metabolites were identified by metabolomic analysis. “Lipids and lipid-like” molecules had the highest proportions. The DEMs in LL1 and ZK1 were mainly categorized 6 and 4 Human Metabolome Database (HMDB) superclasses, respectively. KEGG analysis revealed that the ‘α-linolenic acid metabolism’ was enriched in both LL1 and ZK1. Joint KEGG analysis also revealed that the ‘α-linolenic acid metabolism’ pathway was enriched by both the DEGs and DEMs of LL1. There were 17 DEGs and 8 DEMs enriched in this pathway, and methyl jasmonate (MeJA) may play an important role in the drought response of quinoa. This study will provide information for the identification of drought resistance in quinoa, research on the molecular mechanism of drought resistance, and genetic breeding for drought resistance in quinoa.

## 1. Introduction

As the world’s population continues to grow, so does the demand for food, while global climate change is trending towards warmer temperatures and increased extremes of weather, and a prolonged lack of rainfall and persistent high temperatures have led to severe droughts in many regions, which all lead to abiotic stresses in terms of crop losses and are equivalent to other natural disasters combined [1], posing a great threat to global food security. Climate models predict that these problems will persist and worsen in most parts of the world [2,3].

*Chenopodium quinoa* Willd., commonly known as quinoa, is an annual broad-leaf dicotyledonous plant belonging to the Amaranthaceae family, which originated from the Andean regions and has been cultivated in Peru and Bolivia for more than 7000 years [4,5]. Quinoa is considered to be in the “whole grain” category, which has a higher nutritional level than other grains, containing unique amino acids, carbohydrates, lipids, and micronutrient profiles [6,7]. Since quinoa is gluten-free, these nutrients are well-preserved during its processing [8,9]. In addition, quinoa’s secondary metabolites (including triterpenoids, phenols, β-betaine, and glycine betaine) are also beneficial to human health [9]. Several studies have demonstrated the role of quinoa in promoting child growth, improving celiac disease, and reducing blood lipids [10,11,12].

The unique qualities of quinoa have been utilized to develop a wide range of high value-added products, including frozen food packages, beverages, botanical supplements, cosmetics, breads, and medicines [13,14,15,16]. As quinoa is nutritious and resilient, the FAO declared 2013 as the “International Year of Quinoa”, which has brought quinoa more and more attention and a gradual increase in planting area [17,18]. Quinoa is adapted to many unfavorable climatic conditions, such as drought, high salinity, and frost [19], and can also be grown in geological conditions including lowlands, deserts, and plateaus [20,21]. Drought is the major abiotic stress restricting plant growth and production [22,23]. When subjected to drought, quinoa generates a series of response reactions, and these drought response mechanisms can be categorized into three main groups. (1) Phenotypic changes, including changes in plant height, leaf area, biomass, and root morphology [18]. (2) Physiological and biochemical responses, including photosynthesis, stomatal conductance, antioxidant responses, and osmotic pressure regulation. (3) Molecular-level regulation, including changes in metabolites introduced by altered gene expression.

In addition, some properties make quinoa more drought-resistant. The roots of quinoa are more plastic than those of its close relatives (*C. hircinum* and *C. pallidicaule*), with its taproot growing faster, branching more permanently, and penetrating deeper into the soil under the same drought conditions [24]. Studies on the sub-microscopic structure of quinoa leaves have revealed that calcium oxalate-rich epidermal bladder cells are prevalent on the surface of these leaves, which greatly increase reflectance and reduce transpiration while exerting hygroscopic effects [25,26]. Quinoa also inhibits water loss by reducing its leaf area, increasing its cell wall thickness, and enhancing its cell wall elasticity through the early shedding of old leaves [27]. Based on these characteristics, quinoa can grow in places that receive more than 200 mm of rainfall, but drought still results in lower yields [18,28].

Although the best solution to drought is irrigation, this is difficult to implement in many areas. Therefore, the selection and cultivation of drought-tolerant cultivars to increase yields and meet the growing global demand is an urgent issue for quinoa breeding [29,30]. To achieve this, it is necessary to gain a deeper understanding of the drought response mechanisms in quinoa.

Plant drought tolerance is a complex trait controlled by multiple genes. Studies have shown that the drought responses of quinoa are related to drought intensity and genotype [31,32]. A combined transcriptome and metabolome strategy can better explain the transcriptional regulation of metabolic pathways and has been used in drought tolerance studies on many plants [33,34,35]. In a previous study, the authors screened a drought-tolerant genotype of quinoa and investigated the differences in gene expression levels and metabolites in seedling leaves under drought and re-watering treatments. A total of 38,670 genes and 142 pathways were annotated, and LOC110713661 and LOC110738152 may be the two key genes for drought tolerance in quinoa [36]. These results are useful for the selection of new drought-resistant quinoa cultivars and the identification of drought-tolerant cultivars. However, the study used only one quinoa genotype as a test material.

In this study, we used two quinoa cultivars with different drought resistance, measured their phenotypic, physiological, and biochemical changes under two drought conditions, and conducted joint transcriptomic and metabolomic analyses. The main objectives were as follows: (1) to investigate the phenotypic, physiological, and biochemical differences between two genotypes of quinoa under the same drought conditions; (2) to reveal the key genes and major metabolites affecting the drought resistance of quinoa; and (3) to discover the molecular mechanisms and important genes that play important roles in drought-resistant quinoa cultivars. Our study provides a basis for explaining the molecular basis of drought resistance in quinoa and screening important drought-resistant candidate genes. Our results will provide a basis for the identification and molecular breeding of drought resistance in quinoa.

## 2. Results

### 2.1. Phenotypic Indices

#### 2.1.1. Plant Height (PH) and Leaf Surface Area (LSA)

**Figure 1:**
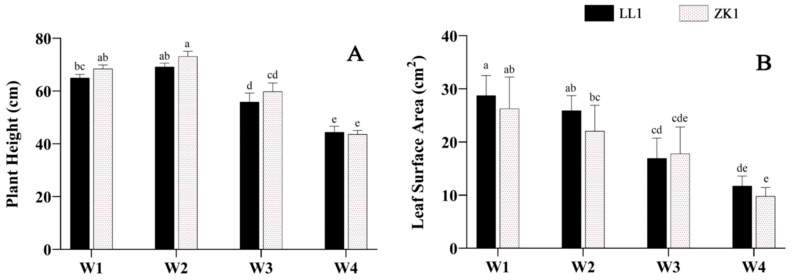
The effects of different drought stress intensities on quinoa seedling height and leaf area. (**A**) Plant height. (**B**) Leaf surface area. Note: W1 represents control group, W2, W3, and W4 represent mild, moderate, and severe drought stress, respectively. The same as below. Vertical bars indicate the mean value ± SD (*n* = 3). The different lowercase letters indicate a significant difference (*p* < 0.05).

With an increase in drought intensity, the PH levels of the two cultivars first increased and then decreased. Under mild stress (W2), the PH increased in all cultivars compared with the control (W1), rising by 6.28% and 6.87%, respectively, while under moderate stress (W3) and severe stress (W4), PH showed a significant decrease, LL1 and ZK1 decreased by 14.23% and 12.38% in W3 (*p* < 0.05) and decreased by 31.75% and 37.00% in W4 (*p* < 0.05), respectively (Figure 1A).

LSA declined with an increasing drought stress and was significantly lower under the W3 and W4 conditions than under W1 (*p* < 0.05) (Figure 1B). LL1 and ZK1 decreased by 42.1% and 32.24% under W3 conditions and by 59.14% and 62.7% under W4 conditions, respectively.

#### 2.1.2. Biomass and Root–Shoot Ratio (RSR)

**Figure 2:**
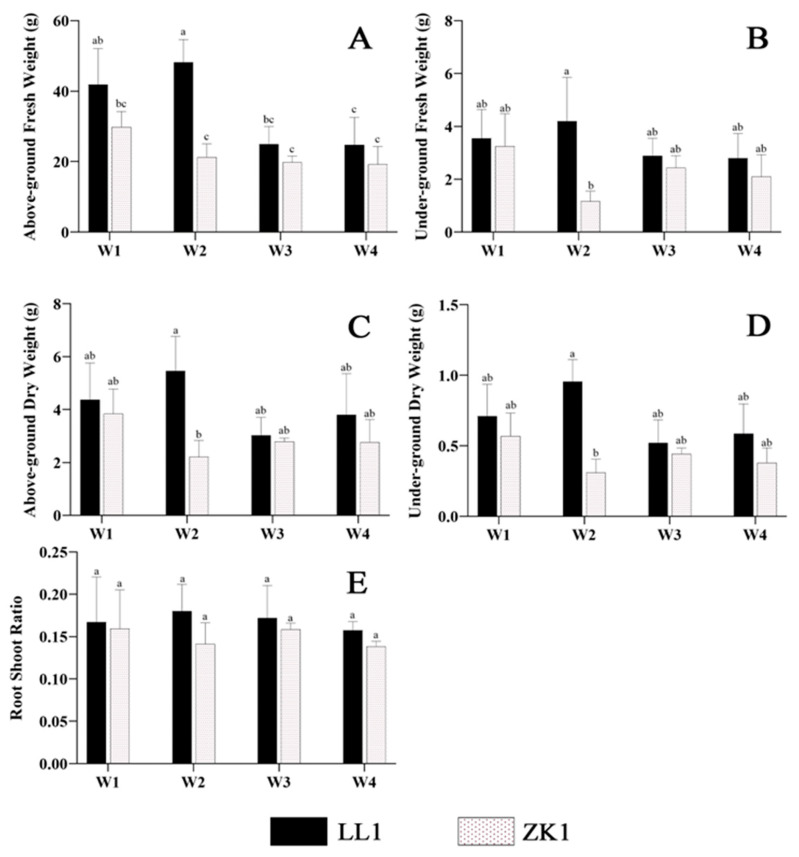
The effects of different drought stress intensities on quinoa biomass and root–shoot ratio. (**A**) The above-ground fresh weight (AGFW). (**B**) The under-ground fresh weight (UGFW). (**C**) The above-ground dry weight (AGDW). (**D**) The under-ground dry weight (UGDW). (**E**) The root–shoot ratio (RSR). Note: vertical bars indicate the mean value ± SD (*n* = 3). The different lowercase letters indicate a significant difference (*p* < 0.05).

Biomass (including AGFW, UGFW, AGDW, and UGDW) (Figure 2A–D) and RSR showed a trend of increasing and then decreasing with an increasing drought stress in LL1 (Figure 2E).

Interestingly, the drought-sensitive cultivar ZK1 showed a decrease in biomass under mild stress (W2), which was significantly lower than that of LL1 (*p* < 0.05). Then, it increased under moderate (W3) and severe stress (W4), but the differences in these changes were not significant (*p* < 0.05).

Although the AGFW and UGFW of both materials varied significantly under different stress intensities, the change trends were generally consistent, resulting in insignificant changes in RSR (Figure 2E).

#### 2.1.3. Relative Water Content (RWC)

**Figure 3:**
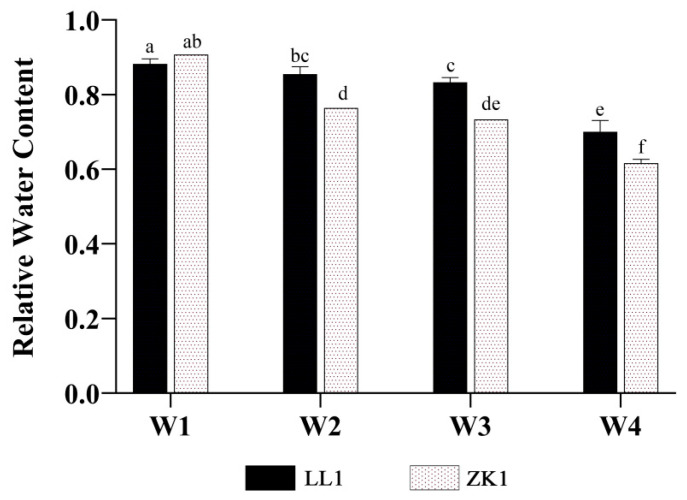
The effects of different drought stress intensities on quinoa relative water content. Note: vertical bars indicate the mean value ± SD (*n* = 3). The different lowercase letters indicate a significant difference (*p* < 0.05).

The RWC of LL1 and ZK1 decreased with an increasing drought stress (Figure 3).

Compared with W1, the RWC of the two quinoa cultivars decreased by 3.09% and 15.77% under W2, decreased by 5.58% and 19.16% under W3, and decreased by 20.60% and 32.06% under W4, respectively. For inter-variety comparison, the differences between LL1 and ZK1 were significant (*p* < 0.05) under W2, W3, and W4.

### 2.2. Physiological and Biochemical Indexes

#### 2.2.1. Root Vigor (Rv)

**Figure 4:**
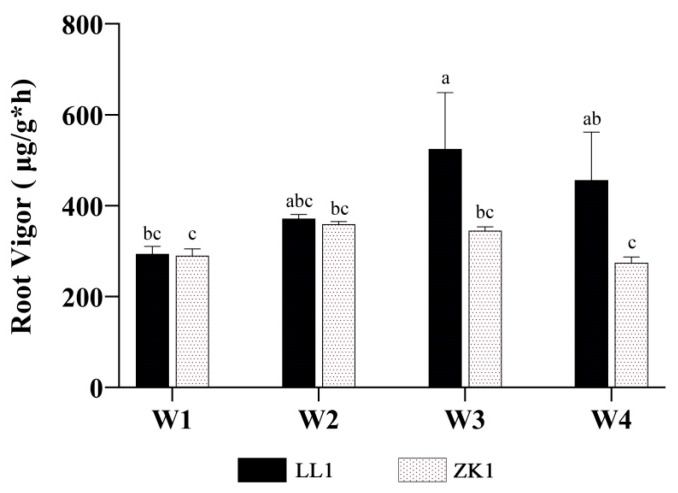
The effects of different drought stress intensities on quinoa root vigor. Note: vertical bars indicate the mean value ± SD (*n* = 3). The different lowercase letters indicate a significant difference (*p* < 0.05).

With an increase in drought stress, the Rv of both LL1 and ZK1 showed a tendency to increase and then decrease (Figure 4), with LL1 and ZK1 reaching their highest values under the W3 (82.1% increase over W1) and W2 (23.9% increase over W1) conditions, respectively.

For inter-variety comparison, the Rv of the two cultivars was not significantly different under W1 conditions, but LL1 was significantly higher than ZK1 under W2 and W3, indicating that LL1 had a higher drought tolerance.

#### 2.2.2. Soluble Sugars and Antioxidase

**Figure 5:**
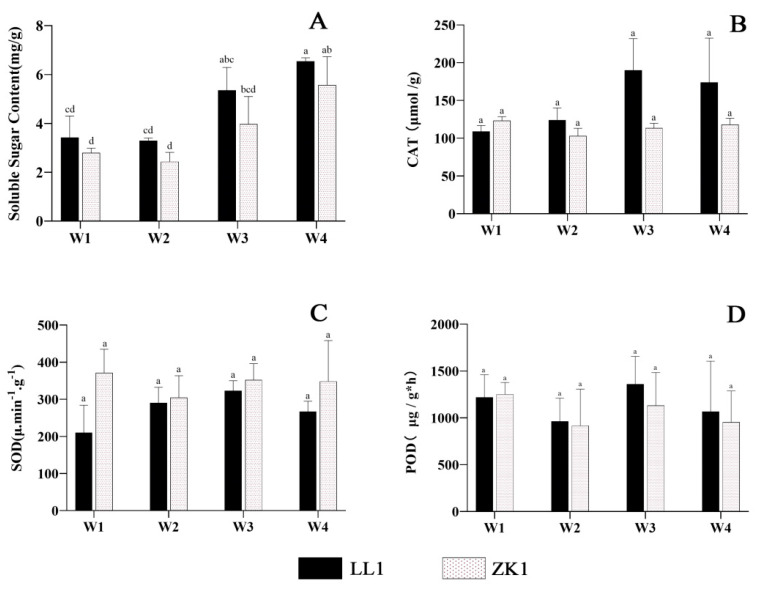
The effects of different drought stress intensities on quinoa soluble sugars and antioxidase. (**A**) The soluble sugar content. (**B**) Enzymatic activity of CAT. (**C**) Enzymatic activity of SOD. (**D**) Enzymatic activity of POD. Note: vertical bars indicate the mean value ± SD (*n* = 3). The different lowercase letters indicate a significant difference (*p* < 0.05).

The soluble sugars in both cultivars decreased to the lowest value under mild stress (W2) (Figure 5A). LL1 and ZK1 decreased by 3.58% and 12.73%, respectively, compared with W1. In W3, they increased by 56.46% and 42.17%, respectively. In the W4 condition, they all reached the highest value, with a significant difference (*p* < 0.05) from W1, and increased by 91.23% and 99.53%, respectively. For inter-variety comparison, the soluble sugars were higher in LL1 than in ZK1 under the three different stress conditions, but these differences were not significant.

The SOD and CAT activities of the two cultivars showed similar trends with an increasing drought stress (Figure 5B,C). Among them, LL1 first increased and then decreased, reaching the highest value in W3. Compared with W1, SOD and CAT increased by 53.7% and 74.5%, respectively. In ZK1, SOD and CAT were first reduced and then increased, and were reduced by 18% and 16.3% under W2 compared with W1, respectively.

POD activity decreased and then increased with an increasing drought intensity (Figure 5D). LL1 reached the highest under W3, which was 11.75% higher than that in W1. LL1 had higher activity than that of ZK1 under different stress intensities.

For inter-variety comparison, SOD/CAT was higher/lower in ZK1 than in LL1 in W2, W3, and W4, but these differences were not significant.

#### 2.2.3. Chlorophyll Content

**Figure 6:**
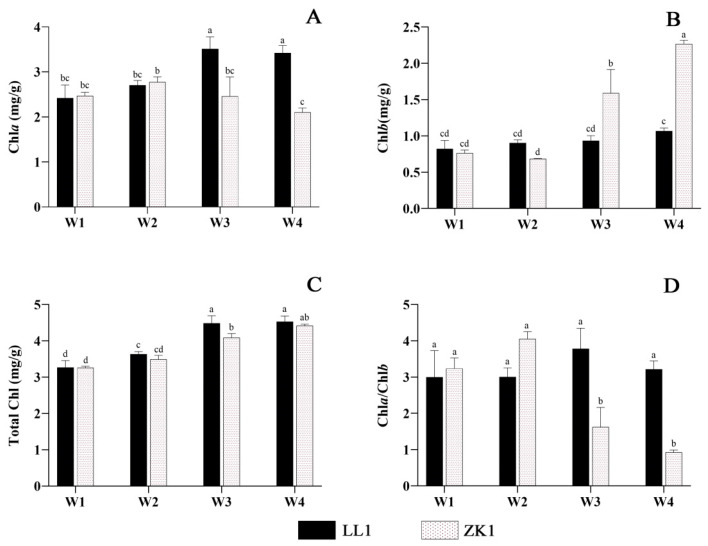
The effects of different drought stress intensities on quinoa chlorophyll. (**A**) Chlorophyll-a content. (**B**) Chlorophyll-b content. (**C**) Total chlorophyll. (**D**) Chlorophyll-a/chlorophyll-b. Note: vertical bars indicate the mean value ± SD (*n* = 3). The different lowercase letters indicate a significant difference (*p* < 0.05).

With an increasing drought stress, Chla and Chla/Chlb content increased and then decreased, while Chlb and total Chl content increased (Figure 6A–C).

For Chla, LL1 reached the highest value in W3, with a significant increase of 45.04% compared with W1. ZK1 reached a maximum under W2 conditions and decreased significantly in W3, W4, and W4 compared with W2. LL1 had significantly higher content than that of ZK1 in both the W3 and W4 conditions.

For Chlb, LL1 increased with an increasing drought stress, but the differences were not significant among the four conditions. In ZK1, after decreasing in the W2 condition, it increased significantly in the W3 and W4 conditions, with contents 108.16% and 196.99% higher than those in W1, respectively. The Chlb content of ZK1 was significantly higher than that of all the other groups in these two conditions. The results showed strong effects of moderate and severe drought stress on the Chlb content of the drought-sensitive variety.

For Chla/b, LL1 and ZK1 reached the highest values under the W3 and W2 conditions, respectively (Figure 6D), with increases of 20.5% and 25.11% compared with W1. LL1 had a significantly higher content than that of ZK1 in the W3 and W4 conditions (*p* < 0.05).

Although the trends of Chla and Chlb were not consistent between the two cultivars under the four drought conditions, the total Chl content underwent the same trend, and both increased with an increasing drought stress. LL1 reached its maximum under W3 (4.485), which was a significant increase of 37.11% compared with W1 (3.27). ZK1 reached its maximum under W4 (4.416), which was a significant increase of 35.5% compared with W1 (3.257).

#### 2.2.4. Photosynthetic Indicators

**Figure 7:**
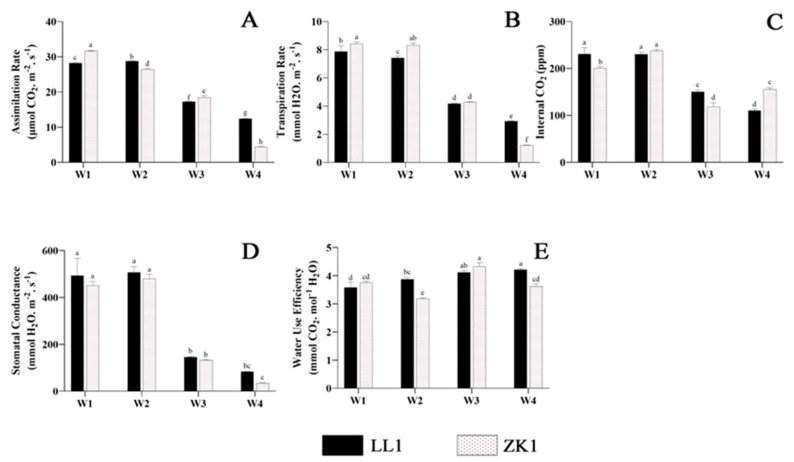
The effects of different drought stress intensities on quinoa photosynthetic properties. (**A**) The assimilation rate. (**B**) The transpiration rate. (**C**) The internal CO_2_ content. (**D**) Stomatal conductance. (**E**) Water use efficiency. Note: vertical bars indicate the mean value ± SD (*n* = 3). The different lowercase letters indicate a significant difference (*p* < 0.05).

The assimilation rate (Pn) of ZK1 continued to decrease with an increasing degree of stress (Figure 7A). The Pn of ZK1 decreased significantly by 16.22%, 41.51%, and 85.99% under the W2, W3, and W4 conditions, respectively. LL1 increased by 2.02% in W2 and then W1 and decreased by 39.07% and 56.07% in W3 and W4, respectively. The Pn values of ZK1 were more influenced by drought stress than those of LL1. Significant differences were observed among all groups.

The transpiration rate (Tr) decreased with an increasing degree of drought stress. Under the W2 condition (Figure 7B), LL1 and ZK1 decreased by 5.83% and 1.19%, respectively. Under the W3 condition, LL1 and ZK1 also decreased significantly (47.02% and 49.23%, respectively). The Tr of both cultivars was reduced to its lowest value under the W4 condition. Notably, the transpiration rate of ZK1 was significantly lower than that of LL1 (*p* < 0.05).

The internal CO_2_ (Ci) of LL1 under the W2 condition was not significantly different from that of W1 (Figure 7C), and it significantly decreased under the W3 and W4 conditions, which were 34.7% and 52.23% lower than that of W1, respectively. ZK1 showed a significant increase (18.4%) in Ci under the W2 condition and a significant decrease (40.8%) under the W3 condition. It increased by 31.17% under the W4 condition compared with W3, but significantly decreased by 22.42% compared with W1. Comparisons between groups showed significant differences between the two cultivars in the three moisture conditions except W2.

The stomatal conductance (Sc) did not change significantly under the W2 condition (Figure 7D), while it decreased significantly under W3 and W4. Both cultivars decreased by 70.5% under W3, while under W4, they decreased by 83.21% and 92.18%, respectively.

With an intensification in drought stress, the water use efficiency (WUE) of LL1 gradually increased and reached its maximum value (4.216) under the W4 condition (Figure 7E), which was 17.56% significantly higher than that of W1. The water utilization rate of ZK1 first decreased and then increased with an intensification in stress, and decreased to its lowest value (3.18) under the W2 condition, which was 32% significantly lower than that of W1, and reached its maximum value under the W3 condition, which was 21% significantly higher than that of W1. A comparison between groups showed that the water utilization of ZK1 was higher than that of LL1 under the W1 and W3 conditions, but this difference was not significant. Under the W2 and W4 conditions, the WUE of LL1 was higher than that of ZK1, and this difference was significant.

### 2.3. Correlation Analysis of Phenotypic, Physiological, and Biochemical Indices

**Figure 8:**
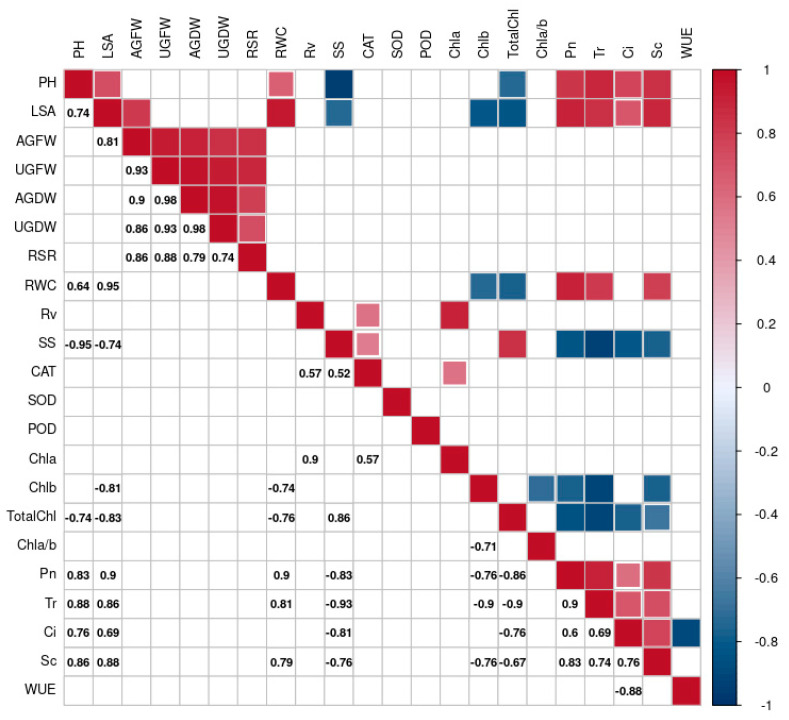
Correlation analysis of physiological and biochemical indicators.

A correlation analysis was performed on the different indicators to reveal their intrinsic linkages using the Corrplot R package, and only the significantly correlated (*p* < 0.05) indicators are shown in Figure 8.

The results showed that PH and LSA were positively correlated (0.74). These two indicators were also negatively correlated with SS (−0.95 and −0.74) and total Chl (−0.74 and −0.83) and positively correlated with four photosynthetic indicators (Pn, Tr, Ci, and Sc), with correlation coefficients above 0.69. All five phenotypic parameters (AGFW, AGDW, UGFW, UGDW, and RSR) were significantly positively correlated with each other, with correlation coefficients above 0.74. Positive correlations were found between SS and total Chl (0.86), and they were negatively correlated with four photosynthetic indicators (Pn, Tr, Ci, and Sc), with correlation coefficients less than −0.81. Chla was positively correlated with Rv, and Chlb was negatively correlated with LSA, Chl a/b, Pn, Tr, and Sc, with correlation coefficients of −0.71, −0.76, −0.9, and 0.76, respectively. Total Chl was negatively correlated with the four photosynthetic indicators, with correlation coefficients less than −0.67. Positive correlations were found between photosynthetic indicators (except Pn), with correlation coefficients greater than 0.6.

### 2.4. Transcriptome Analysis

#### 2.4.1. Sequencing Quality and Alignment Rate

After quality control, transcriptome sequencing obtained a total of 72.03 G clean bases and 254 million clean reads from 12 cDNA libraries. The GC content ranged from 43.43% to 44.21%, and the percentage of Q30 bases was over 92.40% (Table S2).

All samples had more than a 92.11% mapped rate and 75% unique mapped rate, among which TPZ2 had the lowest unique mapped rate (75.51%) and the highest multiple mapped rate (16.60%) (Table S3).

#### 2.4.2. Clustering Heatmap, PCA, and Correlation Analysis

A total of 45,309 genes were obtained from this experiment using the StringTie 1.3.3 and the prepDE.py program (Table S4).

**Figure 9:**
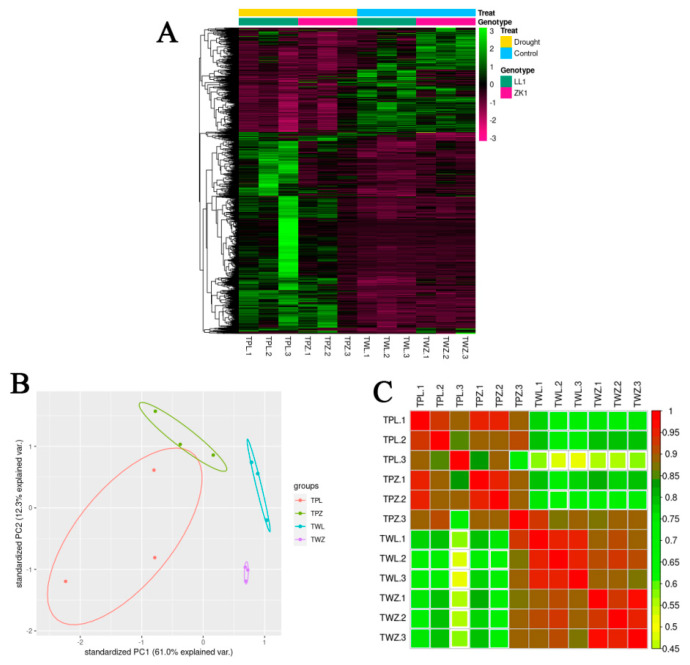

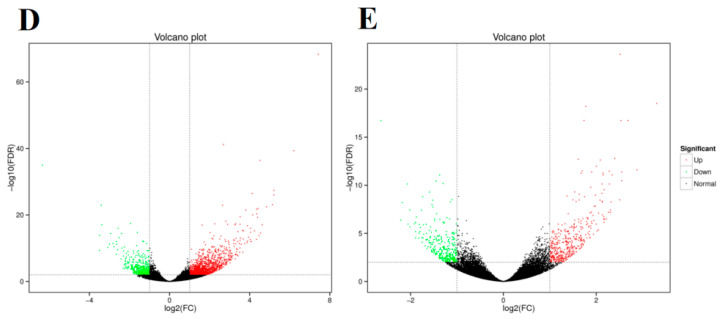
Gene expression clustering, principal components analysis (PCA), and correlation analysis. (**A**) Cluster analysis of gene expression. (**B**) Principle components analysis. (**C**) Correlation analysis. (**D**) LL1 gene expression volcano plot. (**E**) ZK1 gene expression volcano plot.

A clustering heatmap and principal components analysis (PCA) of transcriptomic data revealed a high consistency between three biological replicates of the same treatment for the same sample, whereas this was significantly inconsistent between different treatments for different samples (Figure 9A,B). Correlation analysis showed a high correlation between three replications of the same treatment (correlation coefficient > 0.85) and a low correlation between different treatments of the same material (Figure 9C). These results demonstrate that the transcriptomic data were reliable.

#### 2.4.3. Differentially Expressed Gene Analysis

A differentially expressed genes analysis was conducted using the DESeq2 R package with a threshold of |log_2_(fold changes)| ≥ 1 and Padj < 0.01.

A total of 1756 DEGs (1294 upregulated and 462 downregulated) were obtained in the TWL/TPL comparison group, and 764 DEGs (440 upregulated and 324 downregulated) were obtained in the TWZ/TPZ comparison group (Figure 9D,E; Tables S5 and S6). The Venn diagram showed that there were 316 common DEGs between LL1 and ZK1, which may include important candidate genes for improving the drought tolerance in quinoa (Figure S1).

#### 2.4.4. Gene Ontology Enrichment Analysis

In a GO enrichment analysis, DEGs were classified into the following three categories: biological processes (BPs), cellular components (CCs), and molecular functions (MFs).

**Figure 10:**
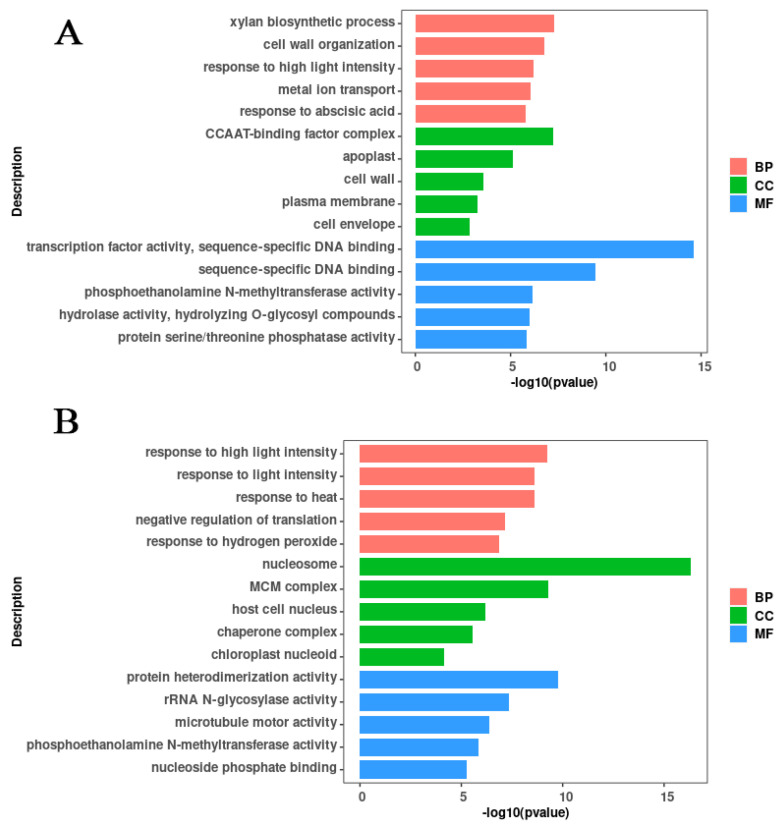
Bar plot for GO enrichment of DEGs. (**A**) GO enrichment bar plot of the LL1 DEGs. (**B**) GO enrichment bar plot of the ZK1 DEGs.

For LL1, 1366 of the 1756 DEGs were enriched into 225 de-abundant GO terms (114 BPs, 21 CCs, and 90 MFs) (Figure 10A; Table S7). In the BPs category, the xylan biosynthetic process (GO:0045492), cell wall organization (GO:0071555), response to high light intensity (GO:0009644), metal ion transport (GO:0030001), and response to abscisic acid (GO:0009737) were the top five most significant subcategories. In the CCs category, CCAAT-binding (G factor complex) (GO:0016602), apoplast (GO:0048046), cell wall (GO:0005618), plasma membrane (GO:0005886), and cell envelope (GO:0030313) were the top five most significant subcategories. In the MFs category, transcription factor activity (GO:0003700), sequence-specific DNA binding (GO:0043565), phosphoethanolamine N-methyltransferase activity (GO:0000234), hydrolase activity, hydrolyzing O-glycosyl compounds (GO:0004553), and protein serine/threonine phosphatase activity (GO:0004722) were the top five most significant subcategories.

For ZK1, 595 DEGs were enriched into 115 de-abundant GO terms (36 BPs, 17 CCs, and 62 MFs) (Figure 10B; Table S8). In the BPs category, response to high light intensity (GO:0009644), response to light intensity (GO:0009642), response to heat (GO:0009408), negative regulation of translation (GO:0017148), and response to hydrogen peroxide (GO:0042542) were the top five most significant subcategories. In the CCs category, nucleosome (GO:0000786), MCM complex (GO:0042555), host cell nucleus (GO:0042025), chaperone complex (GO:0101031), and chloroplast nucleoid (GO:0042644) were the top five most significant subcategories. In the MFs category, protein heterodimerization activity (GO:0046982), rRNA N-glycosylase activity (GO:0030598), microtubule motor activity (GO:0003777), phosphoethanolamine N-methyltransferase activity (GO:0000234), and nucleoside phosphate binding (GO:1901265) were the top five most significant subcategories.

There were 52 common GO terms enriched for the two cultivars, including 19 for BPs, 5 for CCs, and 28 for MFs (Figure S2). In addition, some GO terms directly related to drought stress were enriched in both materials, such as response to water deprivation (GO:0009414), response to abiotic stimulus (GO:0009628), response to stress (GO:0006950), regulation of the abscisic acid biosynthetic process (GO:0010115), and cell wall (GO:0005618).

In addition, some GO terms were enriched only in LL1, such as response to abscisic acid (GO:0009737) and response to osmotic stress (GO:0006970), and it is possible that these mechanisms lead to the stronger drought resistance observed in LL1.

#### 2.4.5. KEGG Enrichment Analysis of Differentially Expressed Genes

A KEGG pathway enrichment analysis was conducted to detect the potential biological functions of DEGs.

**Figure 11:**
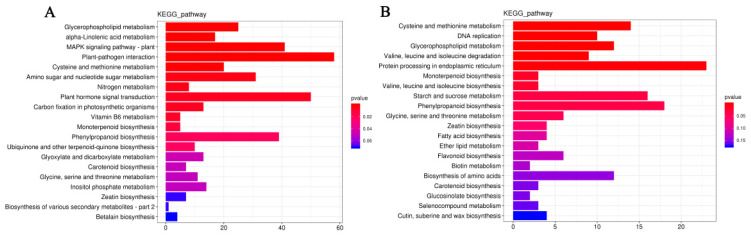
Bar plot for KEGG enrichment of DEGs. (**A**) KEGG enrichment plot of the LL1 DEGs. (**B**) KEGG enrichment plot of the ZK1 DEGs. Note: bar length represents the number of genes.

In the TWL/TPL comparison group, there were 16 significantly enriched pathways, including glycerophospholipid metabolism (ko00564), α-linolenic acid metabolism (ko00592), MAPK signaling pathway—plant (ko04016), plant–pathogen interaction (ko04626), and cysteine and methionine metabolism (ko00270), which were the most top five significant pathways (Figure 11A; Table S9). In the TWZ/TPZ comparison group, there were 10 significantly enriched pathways, including cysteine and methionine metabolism (ko00270), DNA replication (ko03030), glycerophospholipid metabolism (ko00564), valine, leucine, and isoleucine degradation (ko00280), and protein processing in the endoplasmic reticulum (ko04141), which were the top most five significant pathways (Figure 11B; Table S10).

Differentially expressed genes in the two materials were enriched for five common KEGG pathways (Figure S3), including glycerophospholipid metabolism (ko00564), cysteine and methionine metabolism (ko00270), monoterpenoid biosynthesis (ko00902), phenylpropanoid biosynthesis (ko00940), and glycine, serine, and threonine metabolism (ko00260), and there were 25 common genes in these five pathways, with 6, 5, 3, 7, and 4 genes in each pathway, respectively.

**Figure 12:**
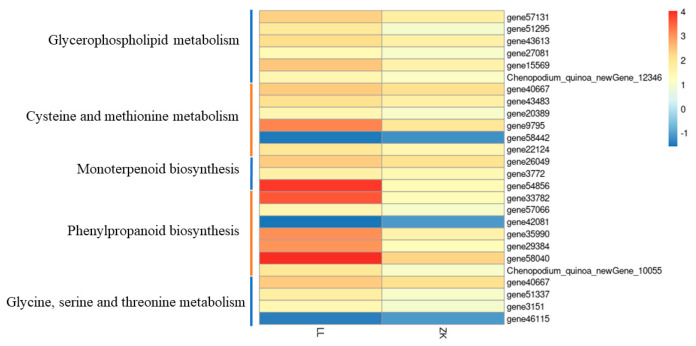
DEGs in the common KEGG pathway.

The heatmap showed that 22 out of these 25 common genes were upregulated, and 3 common genes were downregulated, with a higher |log_2_foldchange| in LL1 than that in ZK1, suggesting that drought-responsive genes were more responsive in LL1 (Figure 12).

#### 2.4.6. RNA-seq Validation by RT–qPCR

To verify the reliability of the RNA-seq results, six DEGs were randomly selected for RT-qPCR analysis. The results showed that the relative expressions of the six genes were consistent with FPKM, and the correlation coefficients ranged from 0.703 to 0.951, indicating the credibility of the transcriptome data (Figure S4).

### 2.5. Metabolome Profiles of the Two Cultivars Under Drought Stress

Metabolite changes were assessed on a qTOF mass spectrometer under default mode. A total of 1844 metabolites were identified in the two cultivars (Table S11). The correlation heatmap showed that samples from the same cultivar were clustered together. The mean correlation coefficients between the six replicates of MWL, MWZ, MPL, and MPZ were 0.97, 0.94, 0.95, and 0.91, respectively (Figure S5). Samples from different cultivars were separated, indicating that the metabolome data were reliable.

#### 2.5.1. PCA and OPLS-DA

The overall metabolic differences among LL1 and ZK1 were preliminarily understood through PCA. The results also showed that six replicates of the same sample clustered together, and different samples/treatments were separated from each other (Figure S6).

For LL1, the PC1 score was 45.44%, and the first two principal components accounted for 62.66% of the total variance (Figure S7A). For ZK1, the PC1 score was 34.98%, and the first two principal components accounted for 54.04% of the total variance (Figure S7B). The treatment and control groups were clearly distinguished in each cultivar, indicating that drought stress affected the metabolic levels in both cultivars.

Orthogonal partial least squares discriminant analysis (OPLS-DA) was used to identify different metabolites. The main parameters of this analysis are R2Y and Q2Y. R2Y represents the goodness-of-fit parameter of the OPLS-DA model, while Q2Y indicates the predictive ability of the model. If both R2Y and Q2Y are closer to one, the model is generally considered more stable and reliable, and if the difference between R2Y and Q2Y exceeds 0.2, this may suggest the presence of more irrelevant model terms or several outlier data points, which could affect the model’s performance and interpretability.

In this study, the Q2Y of the OPLS-DA model for both cultivars was above 0.98, and the difference between R2Y and Q2Y was no more than 0.1, suggesting the excellence of the models (Figure S8).

#### 2.5.2. Differentially Expressed Metabolites (DEMs) Analysis

The variable importance in projection (VIP) value between the metabolites in each comparison group was obtained by OPLS-DA. Metabolites with a VIP > 1, *p*-value < 0.05, and FC ≥ 2 were defined as DEMs.

There were 848 (241 upregulated and 607 downregulated) and 649 (105 upregulated and 544 downregulated) DEMs identified in LL1 and ZK1, respectively (Table S11).

According to a Venn diagram analysis, 481 common DEMs were identified in both LL1 and ZK1, of which 53 DEMs were upregulated, 421 DEMs were downregulated, and 7 DEMs were inconsistent in the two groups (Figure S9). While 367 DEMs (185 upregulated and 182 downregulated) were specifically induced in LL1, 168 DEMs (48 upregulated and 120 downregulated) were specifically induced in ZK1.

#### 2.5.3. Classification of DEMs of HMDB

DEMs were functionally identified using the HMDB.

**Figure 13:**
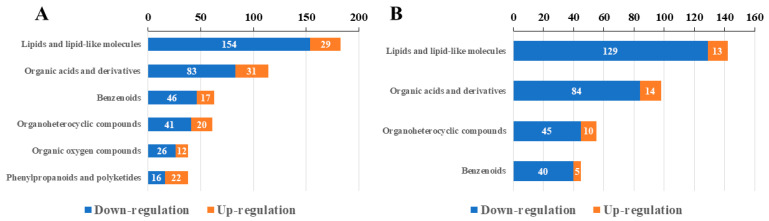
HMDB annotation results. (**A**) HMDB annotation results of LL1. (**B**) HMDB annotation results of ZK1.

The DEMs in LL1 were mainly categorized into the following six HMDB superclasses (>30 DEMs) (Figure 13A): lipids and lipid-like molecules (containing 183 DEMs), organic acids and derivatives (containing 114 DEMs), benzenoids (containing 63 DEMs), organoheterocyclic compounds (containing 61 DEMs), organic oxygen compounds (containing 38 DEMs), and phenylpropanoids and polyketides (containing 38 DEMs). The 183 lipid and lipoid molecular superclasses contained six subclasses (Table S12a), namely, fatty acyls (containing 173 DEMs), prenol lipids (containing 46 DEMs), steroids and steroid derivatives (containing 46 DEMs), glycerophospholipids (containing 12 DEMs), triglycerides (containing 5 DEMs), and sphingomyelins (containing 1 DEM). Most of the DEMs were decreased in the treated group (MPL) compared to the control group (MWL).

The DEMs in ZK1 were mainly categorized into the following four HMDB superclasses (>30 DEMs) (Figure 13B): lipids and lipid-like molecules (containing 142 DEMs), organic acids and derivatives (containing 98 DEMs), organoheterocyclic compounds (containing 55 DEMs), and benzenoids (containing 45 DEMs). The 142 lipid and lipoid molecular superclasses contained five subclasses (Table S12b). Most of the DEMs were decreased in the treated group (MPZ) compared to the control group (MWZ).

#### 2.5.4. Differentially Accumulated Metabolites KEGG Analysis

KEGG annotation and enrichment analyses were performed to further understand the interactions and interregulatory relationships among the DEMs.

**Figure 14:**
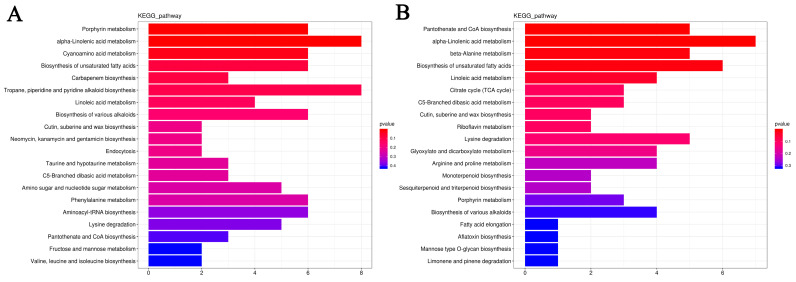
DEMs KEGG enrichment analysis. (**A**) DEMs KEGG enrichment analysis of LL1. (**B**) DEMs KEGG enrichment analysis of ZK1.

For LL1, three pathways were significantly enriched (*p* < 0.05), including porphyrin metabolism, α-linolenic acid metabolism, and cyanoamino acid metabolism (Figure 14A; Table S13). For ZK1, five pathways were significantly enriched (*p* < 0.05), including pantothenate and CoA biosynthesis, α-linolenic acid metabolism, β-alanine metabolism, the biosynthesis of unsaturated fatty acids, and linoleic acid metabolism (Figure 14B; Table S14). Notably, DEMs were significantly enriched in the α-linolenic acid metabolism KEGG pathway in both cultivars, which may play an important role in the drought response of quinoa.

### 2.6. Integrative Transcriptome and Metabolome Analysis

To gain a deeper insight into the drought response mechanism of quinoa, we performed an integrated analysis of the transcriptome and metabolome results. The KEGG pathways significantly enriched (*p* < 0.05) in at least one omics dataset were selected for further analysis.

There were 14 and 11 KEGG pathways enriched in LL1 and ZK1, respectively (Figure S10). A network diagram was generated using Cytoscape. There were six common pathways in LL1 and ZK1 (Figure S11), containing those relating to glycerophospholipid metabolism, cysteine and methionine metabolism, monoterpenoid biosynthesis, phenylpropanoid biosynthesis, glycine, serine, and threonine metabolism, and α-linolenic acid metabolism.

The eight unique KEGG pathways in LL1 (Figure S12) included amino sugar and nucleotide sugar metabolism, nitrogen metabolism, plant hormone signal transduction, carbon fixation in photosynthetic organisms, ubiquinone and other terpenoid-quinone biosynthesis, glyoxylate and dicarboxylate metabolism, carotenoid biosynthesis, and cyanoamino acid metabolism.

The five unique KEGG pathways in ZK1 (Figure S13) included valine, leucine, and isoleucine biosynthesis, starch and sucrose metabolism, pantothenate and CoA biosynthesis, β-alanine metabolism, and linoleic acid metabolism.

The KEGG enrichment analysis above revealed that the DEMs of both LL1 and ZK1 were co-enriched in the α-linolenic acid metabolism (ko00592), so we further analyzed this pathway.

**Figure 15:**
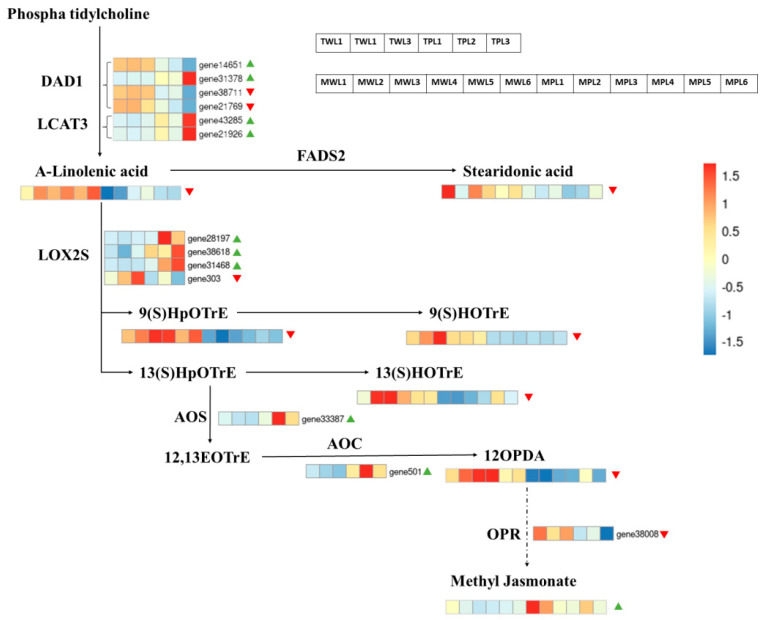
Expressions of DEGs and DEMs in α-linolenic acid metabolism pathway. Heatmap using gene FPKM values and metabolite abundance. Note: Red triangles represent downregulated expression of DEGs and DEMs, green triangles represent up-regulated.

There were 17 genes and 8 metabolites enriched in this pathway (Figure 15).

Of the 17 genes, 6 were downregulated and 11 were upregulated. The top three genes with upregulated multiplicity were gene31378 (triacylglycerol lipase), gene12465 (alcohol dehydrogenase), and gene21566 (alcohol dehydrogenase), with log_2_FC of 2.87, 2.23, and 2.13, respectively; the three genes with the highest downregulated multiplicity were gene303 (linoleate 13S-lipoxygenase 2-1), gene11614 (jasmonate O-methyltransferase), and gene38711 (phospholipase A1-Igamma3), with log_2_FC of −1.63, −1.14, and −1.25, respectively.

Of the eight DEMs, seven were downregulated and one was upregulated. The downregulation of α-linolenic acid content led to the downregulation of a series of downstream products, including 13(S)-HOTrE, α-linolenic acid, 9(S)-HpOTrE, 9(S)-HOTrE, stearidonic acid, traumatic acid, and 12-OPDA, but curiously, the downstream product methyl jasmonate content was upregulated with a log_2_FC of 1.13.

## 3. Discussion

Quinoa originates in the Andean region, is able to adapt to different altitudes and precipitation conditions, and grows with natural rainfall in particularly arid areas [37]. This gives the crop some specific drought tolerance mechanisms [38,39]. Because of this, targeting different genotypes of quinoa will help us to understand the specific drought response mechanisms of quinoa.

### 3.1. Phenotypes, Physiological, and Biochemical Responses

#### 3.1.1. Plant Height and Leaf Area

Under water deficit conditions, plants reduce their transpiration area by reducing their PH. In addition, accelerated root growth also leads to slower stem growth [40]. Several studies have found that drought leads to a decrease in PH [41,42,43]. In quinoa, a study found that deficit irrigation led to a decreased PH, stomatal conductance, and seed yield while increasing the root length density [44]. Another study found that quinoa obtained the highest PH, harvest index, and grain yield at an 80% field water-holding capacity [45]. This study also found that the PHs of two different drought-responsive quinoa were the highest under mild drought stress and then decreased significantly with increasing drought.

LSA is another straightforward drought-related indicator. When plants are subjected to drought, reducing transpiration by decreasing LSA is considered to be an adaptive strategy to reduce water loss [46]. This study found that LSA declined continuously with an intensification in drought stress, and significant declines were observed under moderate and severe stresses.

Correlation analysis revealed that PH was significantly positively correlated with LSA. In addition, PH and LSA were significantly positively correlated with the four photosynthetic indicators (Pn, Tr, Sc, and Ci), possibly due to the fact that, under drought stress, plants reduce transpiration-induced water loss by closing their stomata, and as a trade-off, photosynthetic carbon assimilation decreases, which impacts plant growth [47,48].

#### 3.1.2. Biomass

Under drought stress, plant stomata close, leading to a reduced carbon assimilation and biomass production [49,50]. Huan et al. determined the dry biomasses of two drought-resistant cultivars of quinoa under drought stress and found that the root dry weight increased and the shoot dry weight decreased under drought stress [36]. This study showed that the biomass of LL1 increased under mild drought stress, while that of ZK1 decreased. Under moderate and severe drought stresses, the biomasses of both cultivars decreased, and that of LL1 was higher than that of ZK1 under the same stress conditions. A previous study showed that quinoa can use lower amounts of water per unit of biomass production [51], while the present study demonstrated that there were significant differences among the cultivars. Although the biomasses of the two cultivars differed significantly with an increasing stress, the RSR did not vary significantly among the two cultivars and across the four drought stress conditions, suggesting that drought affects quinoa roots and stems to the same degree. Our results reaffirm that the biomass allocation between the roots and shoot in quinoa is less affected by water deficit [52].

Correlation analysis revealed a significant positive correlation among AGFW, AGDW, UGFW, UGDW, and RSR. Previous studies have emphasized that PH is an important determinant of biomass [53,54], but this study found no significant correlation between them.

#### 3.1.3. Root Vigor

Root is the source of nutrients for the above-ground part of the plant, root traits play an important role in tolerating drought [55], and root vigor is a direct indicator of plant drought tolerance. Under water-limited conditions, soybean improves its water uptake and reduces yield losses by altering its root xylem developmental plasticity [56]. A study showed that, under arid conditions, the Salare ecotype quinoa (grown in dry altiplano) had faster root elongation and a denser depth colonization than the Coastal ecotype (grown in humid coastal lowlands) [57]. In this study, although root morphology was not directly observed, LL1 was found to have a higher root vigor than ZK1. LL1 maintained high levels of root activity under moderate and severe drought stresses.

#### 3.1.4. Soluble Sugar

Osmoregulation is another important mechanism for drought tolerance in plants [52]. As water becomes scarce, plants accumulate sugars such as sucrose, glucose, and fructose in their cells. This osmotic adjustment helps to retain water within plant cells, preventing dehydration and maintaining turgor pressure [58]. In addition, soluble sugars act as antioxidants and participate in the scavenging of ROS [59]. An increase in soluble sugars under drought stress has been reported in other crops [60,61]. Similarly, the soluble sugars in quinoa increased with the intensity of drought stress, indicating the important function of soluble sugars in the response of quinoa to moderate and severe drought stresses.

#### 3.1.5. Reactive Oxygen Species

When plants are subjected to drought stress, osmotic stress induces the accumulation of ROS in cells, which leads to lipid peroxidation and reduces cell membrane integrity. Plants have evolved an antioxidant defense system by producing more antioxidants such as SOD, POD, and CAT that scavenge ROS and mitigate cellular damage [62].

SOD converts harmful O^2−^ to H_2_O_2_ and O_2_, and the H_2_O_2_ can be immediately decomposed into completely harmless water by CAT [63]. This study showed that, under the same drought conditions, the SOD values of a drought-sensitive cultivar were higher than those of a drought-resistant cultivar, while its POD and CAT values were lower than those of the drought-resistant cultivar. The SOD, POD, and CAT values of the drought-resistant cultivar reached their maximum values under moderate stress, and it is possible that moderate drought stress conditions are the maximum intensity that quinoa can withstand. The cells were further damaged and the antioxidant enzyme activities were further affected under severe drought stress.

#### 3.1.6. Chlorophyll

Drought stress may directly affect photosynthesis by destroying cell organelles and disrupting photosynthetic pigments, the chloroplast ultrastructure, and the photosynthetic electron transport chain [64]. Chloroplasts are one of the main targets of abiotic stresses [65]. Chlorophyll content (containing Chla and Chlb) is an important indicator of the photosynthetic capacity of plants. Chla is present in the reaction centers of photosystems I and II and in the pigmented antennae, whereas Chlb is present only in the pigmented antennae system [66].

In this study, the total chlorophyll increased with an intensification in drought, which is consistent with a previous study [67]. However, there was not a significant difference between the two cultivars.

The Chla/Chlb ratio is related to antenna size and is an indicator of functional pigmentation equipment and light acclimatization [68]. A relatively high Chla/Chlb ratio represents highly acclimatized stress in plants. The degree of reduction in Chlb was higher than that in Chla under drought stress in maize, resulting in an increase in the Chla/Chlb ratio [69]. This was presumably due to the faster damage of Chlb compared to Chla under intense stress conditions. Other studies have found that the Chla/Chlb ratio decreased with increased drought stress in Loropetalum chinense [70] and Pinus massoniana [71]. These results indicate that the trends of Chla/Chlb changes in different plants under drought stress are inconsistent.

The present study found significant differences in the Chla/Chlb ratio between the two cultivars. In the drought-resistance cultivar LL1, Chla rose faster than Chlb with an increasing drought intensity, leading to an increase in the Chla/Chlb ratio, whereas these results were reversed in the drought-sensitive cultivar ZK1, which showed a significant decrease in the Chla/Chlb ratio. Similar results were reported in a drought study on Vicia faba [72].

In conclusion, this study showed that, while the total chlorophyll contents of different quinoa cultivars followed the same change trends with increasing drought, there were significant differences in the Chla and Chlb contents between the two cultivars. The Chla/Chlb ratio is a key index for the evaluation of drought tolerance in quinoa.

#### 3.1.7. Photosynthetic Properties

Stomata are channels for gas exchange between plants and the external environment. Under drought conditions, plants limit transpiration by closing their stomata, and a decrease in stomatal conductance leads to lower internal CO_2_ concentrations and less efficient carbon assimilation, and ultimately, plant growth [73,74,75].

Reddy found that highland-type quinoa cultivars have stomata that are insensitive to drought in the early growth stages and instead promote plant water uptake through a high assimilation rate and reduced leaf area, thus helping quinoa to withstand drought stress [76]. González studied the leaf gas exchange and 13C isotopes in 10 different genotypes of quinoa in the arid mountainous region of northwestern Argentina, which receives approximately 160 mm of rainfall during the quinoa growth period, showing that quinoa genotypes with a higher relative leaf stomatal conductance maintained a higher photosynthetic capacity [77].

This study found that mild drought had less effect on quinoa stomata. Under moderate and severe drought stress, the stomatal conductance of quinoa decreased significantly and stomatal closure led to a reduction in the transpiration rate, which also led to a reduction in CO_2_ uptake and a decrease in the assimilation rate. Water use efficiency is an important index for evaluating plant drought tolerance and is equal to the ratio of the assimilation rate and transpiration rate [78]. In this study, since the assimilation rate and transpiration rate decreased simultaneously under moderate and severe drought conditions, the water use efficiency increased instead. This result is consistent with a previous study by Al-Naggar et al., who found that stress treatments applied during the early stages of plant growth increased the WUE, whereas stress treatments applied during the pre-flowering, flowering, and pasty grain phases led to a significant decrease in WUE [79].

### 3.2. Integrated Transcriptome and Metabolome Analysis Reveals Important Metabolic Pathways and Genes

The molecular mechanisms of plant drought resistance are complex, and one promising approach is to integrate high-throughput omics data for bioinformatics analysis to provide a basis for further validation [80]. In this study, we further revealed the molecular mechanism of drought resistance in quinoa through joint transcriptomic and metabolomic analyses.

#### 3.2.1. Two GO Terms Enriched Only in LL1 May Be Associated with Improved Drought Tolerance

GO analysis revealed 52 common GO terms, and some GO terms enriched only in LL1 may be directly related to its drought resistance. The plant hormone abscisic acid (ABA) plays an important role in plant ABA-dependent drought response [81]. Endogenous ABA accumulates in plants under drought stress, which leads to the phosphorylation of SnRK2 by inhibiting the dephosphorylation of PP2C [82,83]. Activated SnRK2 leads to the phosphorylated activation of a series of downstream transcription factors. These transcription factors include SLAC1, which ultimately induces stomatal closure [83,84].

Mild soil drying slightly increased ABA in quinoa xylem, leading to a decrease in the turgor of stomata guard cells, which maintained leaf water potential (ψl) and Amax and resulted in an increase in WUE [21]. In the present study, GO analysis revealed that the two cultivars were co-enriched for some GO terms related to drought resistance. While two GO terms (response to osmotic stress and response to abscisic acid) were only enriched in LL1, this suggests that there may be more ABA accumulation in LL1, which results in a greater antioxidant capacity, leading to its drought resistance. While no ABA-related pathway was enriched in ZK1, there may be other pathways that play a role in its drought response.

#### 3.2.2. Two KEGG Pathways May Play Important Roles in the Drought Response of LL1 and ZK1

Lipids are important structural and functional membrane components that sense extracellular conditions and function as stress mitigators to reduce the intensity of stressors. In addition, components derived from lipids, such as waxes, cuticles, and lignin help to reduce drought stress and tissue damage [85,86,87]. The KEGG analysis in this study revealed that the DEGs in different drought-responsive quinoa cultivars under drought stress were mainly enriched in the “glycerophospholipid metabolism” pathway, which may be related to membrane lipid peroxidation. The metabolite HMDB identification for DEMs also demonstrated the important role of lipids in the drought response of quinoa, with the highest abundance of metabolites in both LL1 and ZK1 belonging to the “lipids and lipid-like molecules” superclass. The levels of all lipid metabolites in both LL1 and ZK1 were reduced under drought stress compared to the control, which is consistent with the results of a drought stress metabolomic study on *Morus alba* L. [88], and this decrease may be caused by membrane damage.

The ascorbate–glutathione cycle plays a key role in H_2_O_2_ detoxification in plants responding to drought stress [89], in which glutathione is synthesized from glutamate, cysteine, and glycine catalyzed by glutathione synthetase [90], and cysteine synthesis is the rate-limiting step in glutathione production [91]. The cysteine and methionine metabolic pathways play important roles in the drought stress response in wheat, oil palm, and Populus ussuriensi [92,93,94]. In the present study, the cysteine and methionine metabolic pathways were enriched by DEGs in both cultivars, with five genes in common, which may be related to cellular antioxidant damage.

### 3.3. Alpha-Linolenic Acid Metabolism Pathway Enriched for Important Drought-Resistant Genes and Metabolites

Since the DEGs and DEMs of LL1 and DEMs of ZK1 were co-enriched in the α-linolenic acid metabolism (ko00592) pathway, we suggest that this pathway is essential for drought tolerance in quinoa. α-linolenic acid in the lipid metabolism is not only a strong antioxidant, but also a precursor for the synthesis of MeJA. Through joint transcriptome and metabolome analyses, Zi et al. revealed that the α-linolenic acid metabolic pathway was significantly enriched under drought stress in maize seedlings. Maize adjusts the fatty acid abundance under drought stress in an effort to maintain membrane integrity and a suitable fat metabolism route [95]. This study also found that the α-linolenic acid metabolism (ko00592) was enriched by both DEGs and DEMs in the drought-tolerant quinoa variety, which may play an important role in the drought response of quinoa.

Plant methyl jasmonate (MeJA), as a hormone involved in plant signaling, is an important cellular regulator that plays a vital role in plant development processes and mediates defense responses to a variety of abiotic stresses [96]. MeJA modulates defense-related antioxidant enzyme activities, induces the accumulation of defense compounds (e.g., phenolic compounds), and regulates photosynthesis, such as stomatal closure [97].

Several studies have shown that the exogenous addition of MeJA is effective in improving the drought tolerance in barley (*Hordeum vulgare* L.) [55], wheat (*Triticum aestivum* L.) [98,99,100], soybean (Glycine max) [101,102], sugar beets (*Beta vulgaris* L.) [103], tomato (*Solanum lycopersicum* L.) [104], and rice (*Oryza sativa* L.) [105]. Drought conditions also endogenize MeJA accumulation, and a synergistic effect may exist between ABA and MeJA under drought conditions [106,107].

In our study, we found that MeJA was upregulated in drought-responsive cultivars under drought stress, with a multiplicity of upregulation of 2.47. Our study found that, while some upstream compounds were downregulated in drought-stressed and drought-resistant cultivars, curiously, the end product MeJA was upregulated.

We hypothesize that α-linolenic acid mostly participates in the synthesis of JA during seedling drought and that MeJA may play an important role in the drought response of quinoa.

## 4. Materials and Methods

### 4.1. Plant Materials

This study was conducted in July 2021 under controlled conditions at the Plant Physiology Laboratory of Jilin Normal University (Siping, China). Two quinoa cultivars, LL1 (drought-tolerant) and ZK1 (drought-sensitive), with significant differences in drought resistance, were selected according to our pre-experiments. Seeds of LL1 were provided by the Gansu Academdemy of Agricultural Sciences, and seeds of ZK1 were provided by the Institute of Crop Research, Chinese Academy of Agricultural Sciences.

### 4.2. Seed Germination and Seedling Growth Performance

Seeds with a uniform size and no pests or diseases were sterilized with 10% (*v*/*v*) NaClO for 15 min, rinsed three times, and immersed in distilled water for 6 h. Then, the seeds were sown evenly in plastic pots (bottom diameter × upper diameter × height = 13 cm × 19 cm × 16 cm) covered with 5 cm of nutrient soil (nutrient soil and perlite mixed at a ratio of 3:1). Each pot was planted with 20 seeds for a total of 20 pots per cultivar.

Then, all pots were cultivated in a light incubator. The light intensity was controlled with a 16 h photoperiod of 6000 lux. The temperature was controlled at 26 ± 1 °C/20 ± 1 °C (day/night), and the relative humidity was controlled within 55–65%. To ensure the normal growth of the plants, 200 mL of water was added to each pot every day, and Hoagland nutrient solutions were added once a week.

The seedlings were interplanted twice at the four- and eight-true leaf stages, and finally, 10 seedlings with a uniform growth were left in each pot.

### 4.3. Drought Stress Treatments of Different Intensities

Twenty-eight days after sowing, 12 pots of each cultivar were randomly divided into four groups, and each group contained 3 pots for three replications. The seedlings were rinsed with sterilized distilled water and placed into different concentrations of PEG-6000 to simulate drought stress. The control group (W1) was watered with only 200 mL of Hoagland nutrient solution, and the treatment groups used 25% (*w*/*v*), 30% (*w*/*v*), and 35% (*w*/*v*) PEG-6000 to simulate mild (W2), moderate (W3), and severe (W4) drought stresses, respectively. The Hogland nutrient solution was replenished at 8:00 a.m. every day to ensure that the concentration of PEG-6000 remained constant. After 10 days of stress, physiological and biochemical indicators were measured.

#### 4.3.1. Determination of Plant Height and Leaf Surface Area

After 10 days of stress, five seedlings were randomly selected from each pot to measure their plant height (PH) using a meter scale. The leaf surface area (LSA) was measured on the 4th leaf from the bottom of each plant using a leaf area meter (YMJ-B, TOP Holding Co., Ltd., Hangzhou, Zhejiang, China).

#### 4.3.2. Determination of Biomass and Root–Shoot Ratio

The roots of each plant were cut using scissors, and the above-/under-ground fresh weights (AGFWs/UGFWs) were weighed directly using an electronic balance (Sartorius ME254S, Sartorius AG, Gottingen, Germany). Then, they were placed in a blast drying oven at 105 °C for 15 min. They were dried at 80 °C until they reached a constant weight and the above-/under-ground dry weights (AGDWs/UGDWs) were weighed. The root–shoot ratio (RSR) was calculated as the UGFW divided by the AGFW.

#### 4.3.3. Determination of Leaf Relative Water Content

The leaf relative water content (RWC) was determined by weighing. Three plants per treatment were randomly selected for sampling, and the 4th leaf from the bottom was selected from each plant. The leaf fresh weight (FW) was weighed directly using an electronic balance. Then, the leaves were soaked in distilled water for 6 h, drained on filter paper, weighed to the turgid weight (TW), and then dried to a constant weight at 105 °C to determine the dry weight (DW). The calculation formula is as follows:

(1) $$RWC=\frac{FW-DW}{TW-DW}\times 100\%$$

#### 4.3.4. Determination of Root Vigor

Root vigor was determined by the colorimetric method of triphenyl tetrazolium chloride (TTC) [108].

A total of 0.5 g of fresh root tip was immersed in an equal volume of TTC and phosphate buffer (pH 7.5) and reacted in darkness at 37 °C for 3 h. The reaction was terminated by adding 2 mL of H_2_SO_4_ (1 mol/L). The roots were removed and ground in a mortar with 5 mL of ethyl acetate and quartz sand. The red extract was transferred to a 10 mL volumetric flask, and the residue was extracted with ethyl acetate three times. The volume was adjusted to 10 mL, and the absorbance value was measured using a UV spectrophotometer at 485 nm. The root vigor was calculated as the reduction in TTC per gram of root in 1 h (μg·g^−1^ h^−1^).

#### 4.3.5. Determination of Leaf Soluble Sugars and Antioxidant Enzyme Activities

The soluble sugar content was determined according to the anthrone colorimetric method [109]. For the extraction of leaf enzyme solution, 1 g of fresh leaves of each treatment was taken and ground in liquid nitrogen. Then, 5 mL of PBS phosphate buffer (pH 7.8, 1% PVP) was added, centrifuged for 15 min (12,000 r/min, 4 °C), and the supernatant was collected. The protein concentration was determined by using Coomassie brilliant blue G-250 staining for antioxidant enzyme activity determination. Catalase (CAT, EC 1.11.1.6) activity was determined by the ultraviolet absorption method [110]. Superoxide dismutase (SOD, EC 1.15.1.1) activity was determined by the nitrogen blue tetrazolium (NBT) method [111]. Peroxidase (POD, EC 1.11.1.7) activity was determined by the guaiacol method [112].

#### 4.3.6. Determination of Leaf Chlorophyll Content

The leaves of each treatment were sampled and dried at 80 °C overnight. Then, 30 mg was weighed and ground in liquid nitrogen. Then, 6 mL of 95% (*v*/*v*) ethanol was added and left in the dark for 48 h. The supernatant was collected after centrifugation for 5 min (12,000 r/min 4 °C). The chlorophyll concentrations (Chla and Chlb) were measured using a spectrophotometer at 649 nm and 665 nm. The chlorophyll content was calculated as follows:

(2) $$Chla\;(mg/g^{-1}FW)=\frac{(13.95\times OD665-6.88\times OD649)\times 0.006}{0.03}$$

(3) $$Chlb\;(mg\cdot g^{-1}FW)=\frac{(24.96\times OD649-7.32\times OD665)\times 0.006}{0.03}$$

(4) $$Total\;Chl\;\left(mg\cdot g^{-1}FW\right)=Chla+Chlb$$

#### 4.3.7. Determination of Photosynthetic Properties

Three plants with a uniform growth were randomly selected from each treatment for the determination of the photosynthetic parameters. The fourth leaf from the bottom was used to determine the assimilation rate (Pn), transpiration rate (Tr), intercellular CO_2_ concentration (Ci), and water use efficiency (WUE) using a portable photosynthesis system (PP SYSTEMS CIRAS-3, Amesbury, MA, USA) between 8:00 and 11:00 a.m.

### 4.4. Transcriptome and Metabolic Analysis

#### 4.4.1. Samples Collection

After 10 days of stress, a metabolomic analysis was performed on seedlings from the control (W1) and moderate stress treatments (W3). The third leaf from the bottom of each seedling was sampled, rinsed with distilled water and blotted dry on filter paper, snap-frozen in liquid nitrogen, and stored at −80 °C. Three biological replicates were performed for transcriptome analysis, and six biological replicates were performed for metabolome analysis. A total of 12 and 24 samples were collected for the transcriptome and metabolome analyses, respectively (Table 1).

#### 4.4.2. RNA Extraction, Library Construction, and Sequencing

The total RNA was extracted using Trizol reagent (Tiangen DP411, Beijing, China) following the manufacturer’s instructions. The purity and concentration of the RNA were measured using a NanoDrop 2000 (Thermo Fisher Scientific Inc., Waltham, MA, USA), and its integrity was assessed using an Agilent 2100 bioanalyzer (Agilent Technologies, City of Santa Clara, CA, USA). The cDNA library construction, library quality assessment, and sequencing were performed by Biomarker Technologies (Beijing, China). Clean reads were aligned to the quinoa reference genome (https://www.ncbi.nlm.nih.gov/genome/?term=quinoa (accessed on 6 January 2024)) using the HISAT2 software (https://ccb.jhu.edu/software/hisat2/index.shtml (accessed on 6 January 2024), v2.0.4). The gene relative abundances were measured in read counts using the StringTie software (https://ccb.jhu.edu/software/stringtie/ (accessed on 7 January 2024), v1.3.3) with the “merge” option. Raw counts were calculated using the Python script “prepDE.py”.

#### 4.4.3. Gene Annotation, Differential Expression, and Enrichment Analysis

Genes were annotated according to several available protein databases, including Nr (NCBI non-redundant protein sequences), Nt (NCBI non-redundant nucleotide sequences), Pfam (Protein family), KOG/COG (Clusters of Orthologous Groups of proteins), Swiss-Prot (a manually annotated and reviewed protein sequence database), KO (KEGG Ortholog database), and GO (Gene Ontology).

The read counts were then normalized to fragments per kilobase of transcript per million mapped reads (FPKMs). PCA was conducted using the “prcomp” function in R, plotting was performed using the ggbilot R package (http://github.com/vqv/ggbiplot, (accessed on 15 January 2024)), clustering analysis was performed using the Pheatmap R package (https://CRAN.R-project.org/package=pheatmap (accessed on 15 January 2024)), and correlation analysis was performed using the Corrplot R package (https://github.com/taiyun/corrplot (accessed on 15 January 2024)). Differentially expressed genes analysis was processed by the DESeq2 R package (https://bioconductor.org/packages/DESeq2, (accessed on 15 January 2024)). The criteria for differentially expressed genes were set as |log_2_(fold changes)| ≥ 1 and Padj < 0.01. GO and KEGG enrichment analyses were performed using the ClusterProfiler R package (https://github.com/GuangchuangYu/clusterProfiler, (accessed on 15 January 2024)). ReviGO (http://revigo.irb.hr, (accessed on 15 January 2024)) was conducted to remove redundant GO terms by finding a representative subset of the GO terms.

#### 4.4.4. RT-qPCR Analysis

To validate the accuracy of the transcriptome sequencing results, six DEGs were selected randomly from all DEGs for a quantitative real-time PCR (RT-qPCR) analysis. The primer sequences are shown in Table S1. The first-strand cDNA was synthesized by the TUREscript 1st Stand cDNA Synthesis Kit (Aidlab Biotech, Beijing, China). The RT-qPCR assay was performed with 2 × SYBR^®^ Green Premix (TaKaRa Biotechnology, Dalian, China) on a qTOWER2.2 real-time quantitative PCR system (Analytik Jena, Jena, Germany). ACT-1 was used as an endogenous housekeeper gene (Table S1). The relative gene expression was calculated using the 2^−ΔΔ^Ct method. Each experiment was conducted in three replicates.

#### 4.4.5. Metabolic Extraction and Analysis

UPLC-OTOF-MS was performed on a Waters Xevo G2-XS QTOF. Metabolites were extracted from each leaf sample (approximately 50 mg). Raw data obtained by UPLC–MS were collected and processed using the MassLynx (Waters Corporation, Milford, MA, USA) and Progenesis QI software 3.0 (Nonlinear Dynamics, Newcastle, UK). PCA and Spearman correlation analyses were used to judge the repeatability of the samples within the group. The identified compounds were searched for their classification and pathway information in the HMDB (http://www.hmdb.ca (accessed on 28 January 2024)) and KEGG (http://www.kegg.jp/, (accessed on 15 January 2024)) databases. A *t*-test was used to calculate the *p* value of each compound. The ropls R package (https://www.bioconductor.org/packages/devel/bioc/vignettes/ropls/inst/doc/ropls-vignette.html (accessed on 28 January 2024)) was used to perform OPLS-DA modeling, and 200 permutation tests were performed to verify the reliability of the model. The VIP value of the model was calculated using multiple cross-validation. The method of combining the difference multiple, the *p* value, and the VIP value of the OPLS-DA model was adopted to screen the differentially abundant metabolites. Metabolites with a variable importance in projection (VIP) > 1, fold change (FC) > 1 and *p* value < 0.05 were considered to be differentially expressed metabolites (DEMs). The differentially accumulated metabolites with KEGG pathway enrichment significance were calculated using a hypergeometric distribution test.

#### 4.4.6. Multi-Omics Integration Analysis

To obtain the intrinsic linkages of differentially expressed genes and differentially accumulated metabolites, we performed a joint analysis of the two cultivars. DEGs or DEMs that were significantly enriched in a particular KEGG pathway were considered to be important pathways for further analysis.

### 4.5. Data Statistics

Physiological data were organized using WPS Office, SPSS 19.0 (https://www.ibm.com/spss (accessed on 28 December 2023)) for one-way ANOVA (one-way statistical analysis of variance) and the least significant difference method (Tukey method) for multiple comparisons, with a significant difference defined as *p* < 0.05. All data are expressed as mean ± standard error (SE).

## 5. Conclusions

Drought is one of the important abiotic stress factors affecting crop yield. In this study, we determined the phenotypic, physiological and biochemical indices of two different drought-resistant quinoa cultivars under drought stress, and performed transcriptomic and metabolomic analyses. The results indicated that the Chla/Chlb ratio may be critical for drought tolerance in quinoa. DEGs were mainly enriched in the “glycerophospholipid metabolism” and “cysteine and methionine metabolic” pathways, which may play important roles in quinoa under drought stress; DEMs mostly belonged to lipids and lipid-like molecules. Joint analyses revealed that “α-linolenic acid in lipid metabolism” was an important metabolic pathway in LL1, and a total of 17 genes and 8 metabolites were enriched in this pathway.

Our results provide new insights for the identification of drought resistance in quinoa, the study of molecular mechanisms of drought tolerance, and the utilization of important drought resistance genes.

## Figures and Tables

**Table 1:** Grouping and numbering of metabolome samples.

Plant Material	Treatment	RNA-seq Samples	GC-MS Samples
LL1	Control	TWL1, TWL2, TWL3	MWL1, MWL2, MWL3, MWL4, MWL5, MWL6
	Drought	TPL1, TPL2, TPL3	MPL1, MPL2, MPL3, MPL4, MPL5, MPL6
ZK1	Control	TWZ1, TWZ2, TWZ3	MWZ1, MWZ2, MWZ3, MWZ4, MWZ5, MWZ6
	Drought	TPZ1, TPZ2, TPZ3	MPZ1, MPZ2, MPZ3, MPZ4, MPZ5, MPZ6

## Data Availability

The Illumina raw sequencing profiles were submitted to the NCBI. BioProject data-base under number PRJNA1041942.

## Supplementary Materials

The supporting information can be downloaded at: https://www.mdpi.com/article/10.3390/ijms252212188/s1.

## Abbreviations

PH	plant height
LSA	leaf surface area
AGFW	above-ground fresh weight
AGDW	above-ground dry weight
UGFW	under-ground fresh weight
UGDW	under-ground dry weight
RSR	root–shoot ratio
RWC	relative water content
Rv	root vigor
SS	soluble sugar
Pn	assimilation rate
Tr	transpiration rate
Sc	stomatal conductance
Ci	internal CO_2_
WUE	water use efficiency
DEGs	differentially expressed genes
DEMs	differentially expressed metabolites
GO	Gene Ontology
KEGG	Kyoto Encyclopedia of Genes and Genomes
BP	biological processes
CC	cellular components
MF	molecular functions
PCA	principal component analysis
OPLS-DA	orthogonal partial least squares discriminant analysis
