# Supplementary figures and images for: Integrating Physiology, Transcriptome, and Metabolome Analyses Reveals the Drought Response in Two Quinoa Cultivars with Contrasting Drought Tolerance
Source: Int J Mol Sci. 2024 Nov 13;25(22):12188. doi: 10.3390/ijms252212188 (PMC11594460; doi:10.3390/ijms252212188)

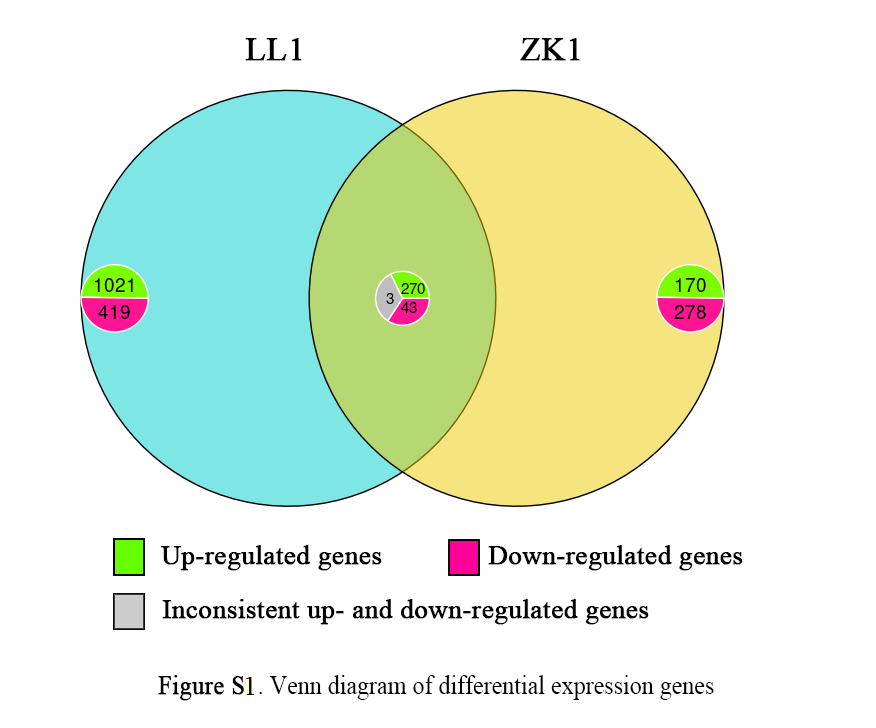

Supplement: Supplementary file 1 [file ijms-25-12188-s001.zip › Figure S1 DEGsVenn.jpg]

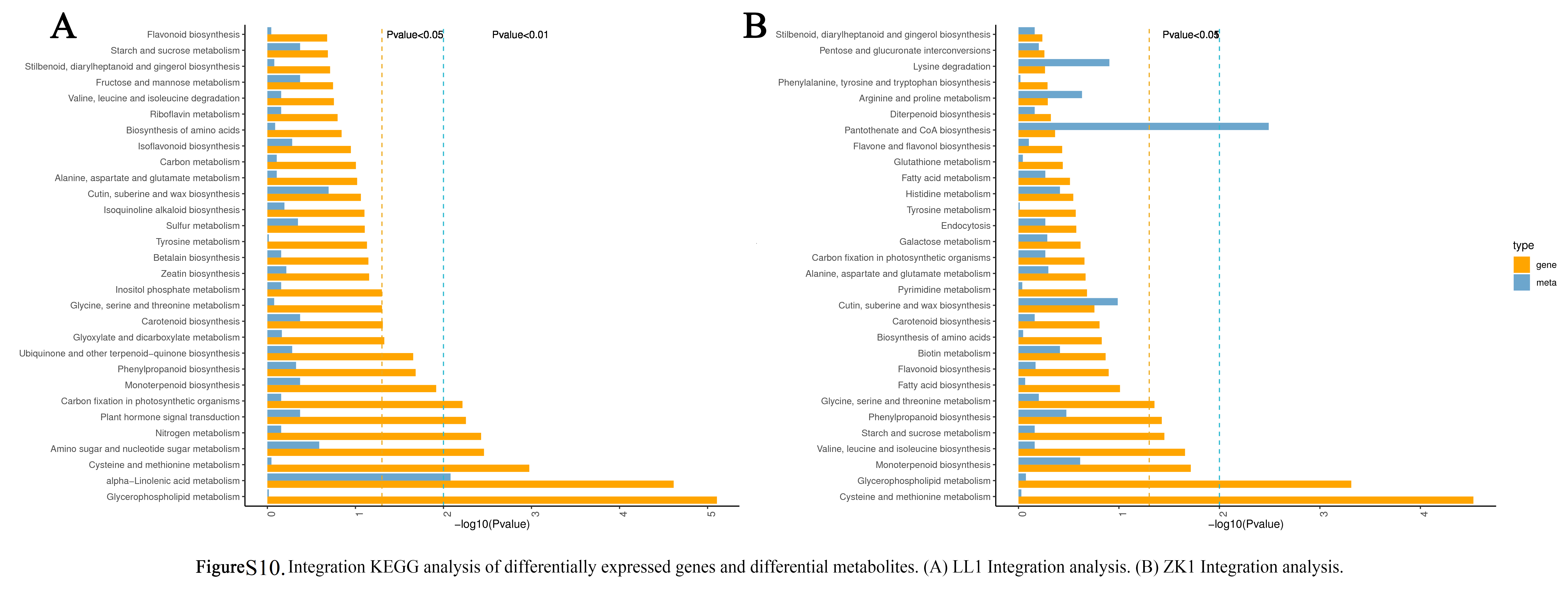

Supplement: Supplementary file 1 [file ijms-25-12188-s001.zip › Figure S10 Integration KEGG analysis.tif]

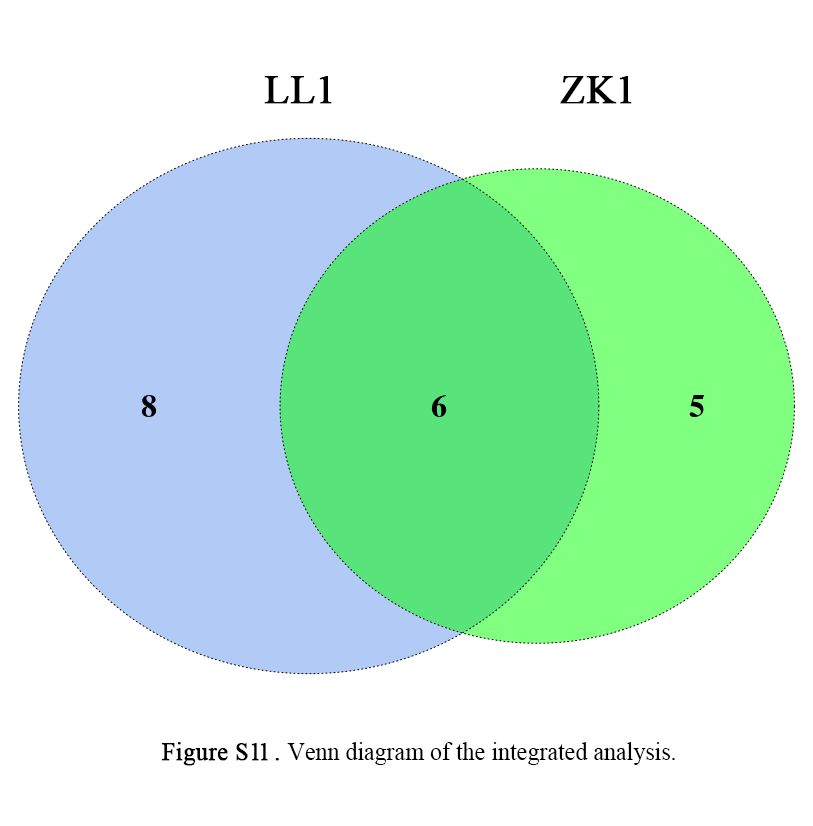

Supplement: Supplementary file 1 [file ijms-25-12188-s001.zip › Figure S11 CombinedKeggLvsZ.tif]

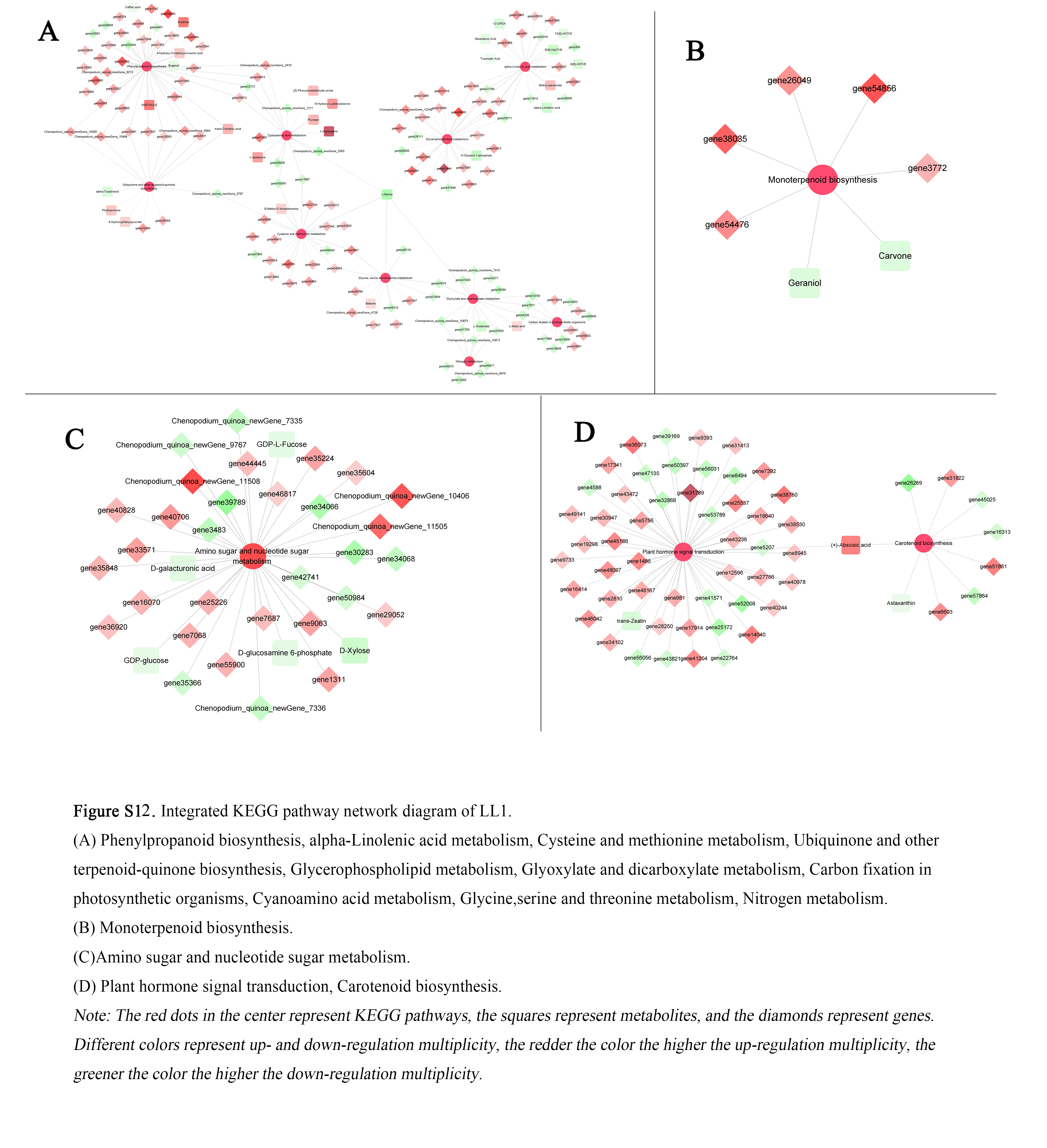

Supplement: Supplementary file 1 [file ijms-25-12188-s001.zip › Figure S12 Integrated KEGG pathway network diagram of LL1s.tif]

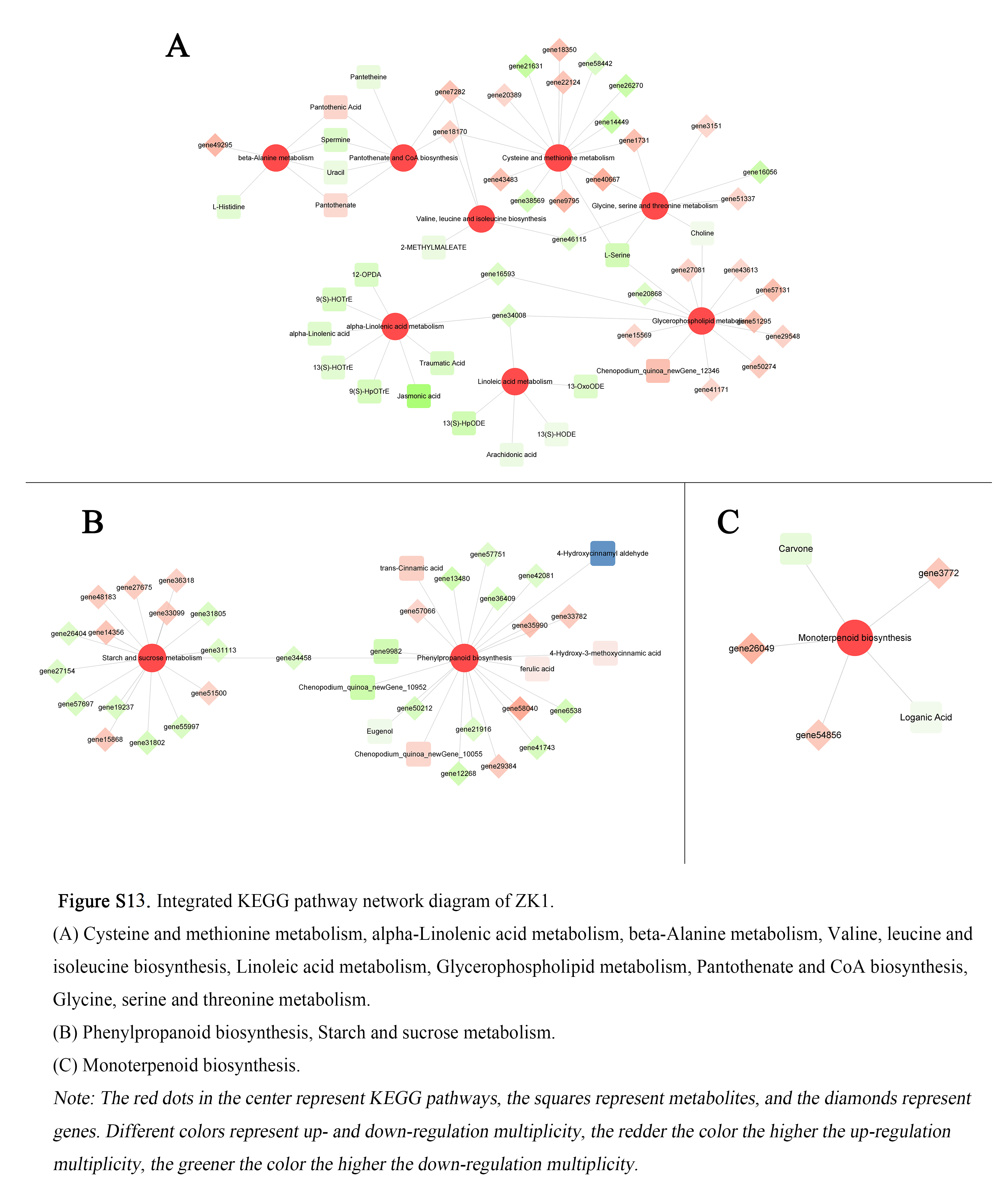

Supplement: Supplementary file 1 [file ijms-25-12188-s001.zip › Figure S13 Integrated KEGG pathway network diagram of ZK1s.tif]

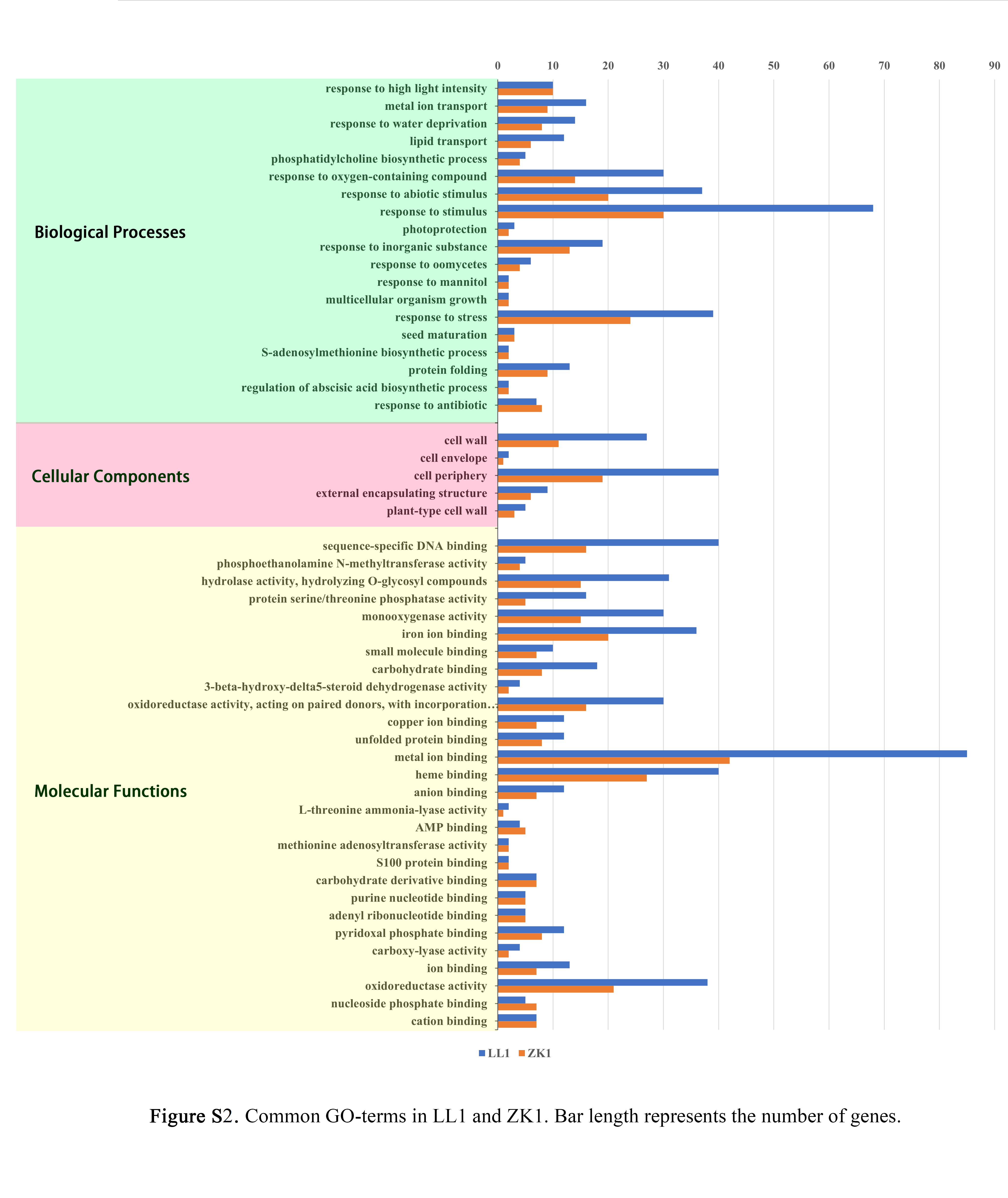

Supplement: Supplementary file 1 [file ijms-25-12188-s001.zip › Figure S2 CommonGO-terms.tif]

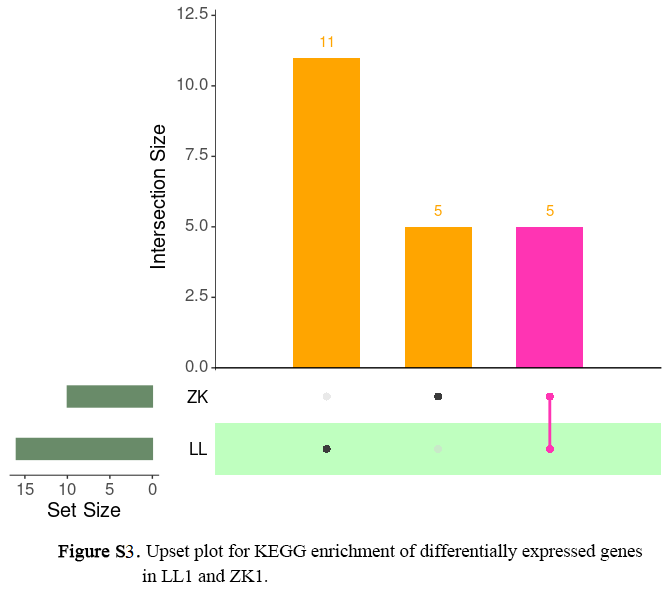

Supplement: Supplementary file 1 [file ijms-25-12188-s001.zip › Figure S3 CommonDEGKeggUpsetR.tif]

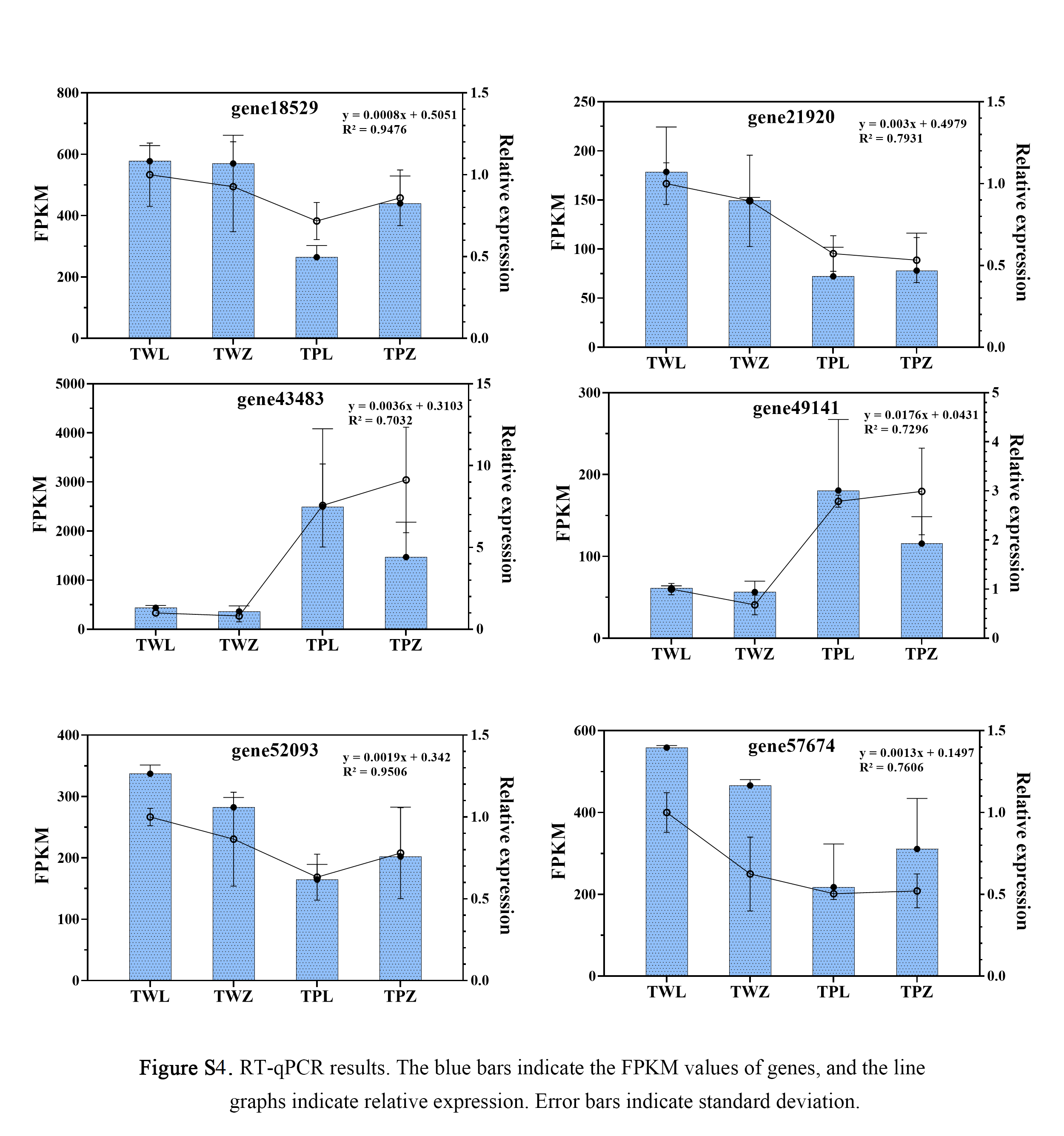

Supplement: Supplementary file 1 [file ijms-25-12188-s001.zip › Figure S4 RT-qPCR.tif]

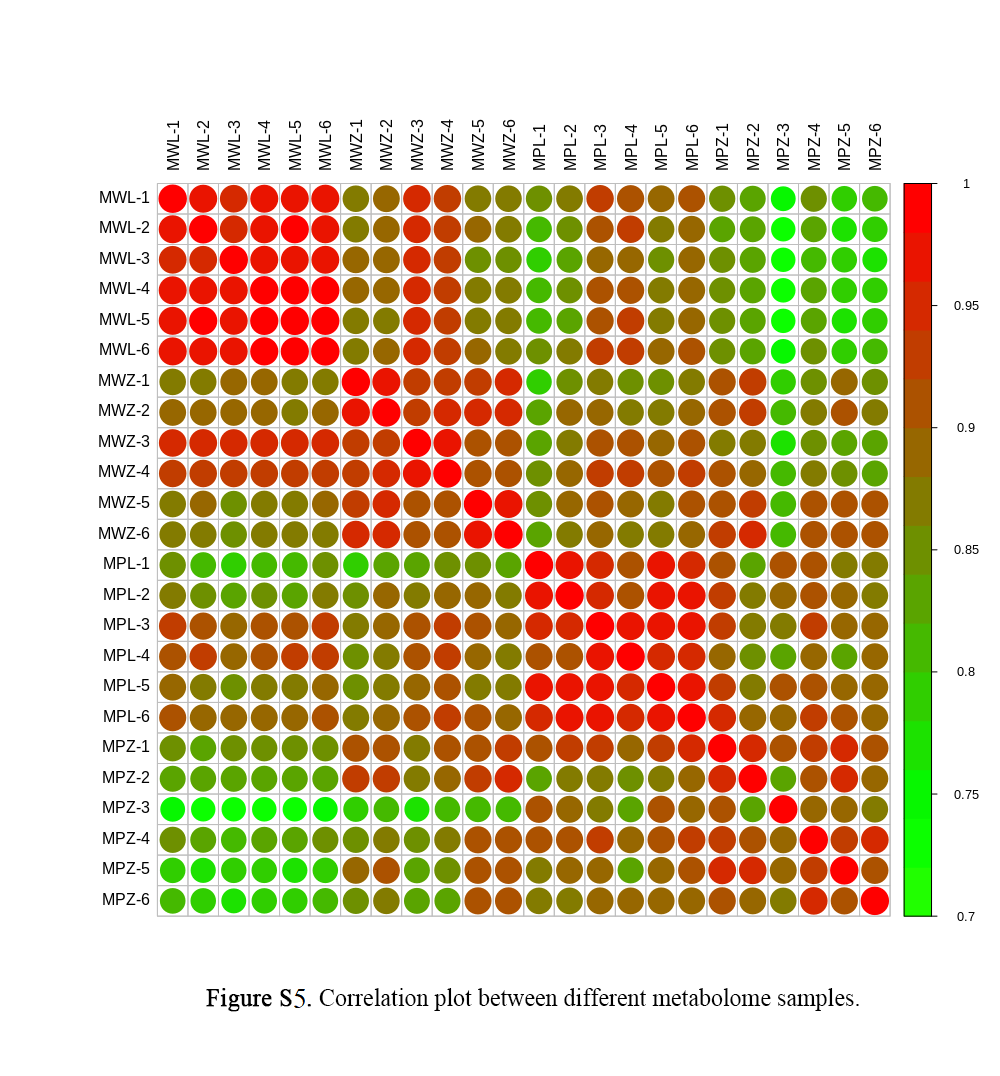

Supplement: Supplementary file 1 [file ijms-25-12188-s001.zip › Figure S5 MetaCorrplot.tif]

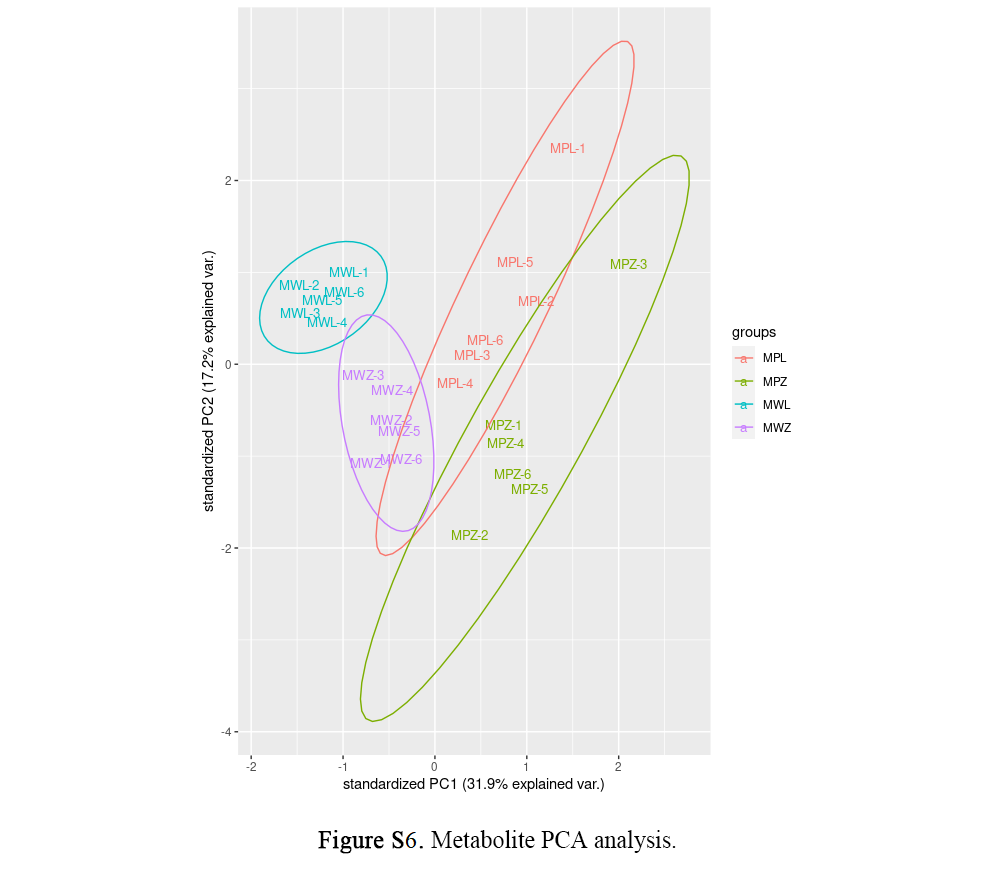

Supplement: Supplementary file 1 [file ijms-25-12188-s001.zip › Figure S6 Metabolite PCA analysis.tif]

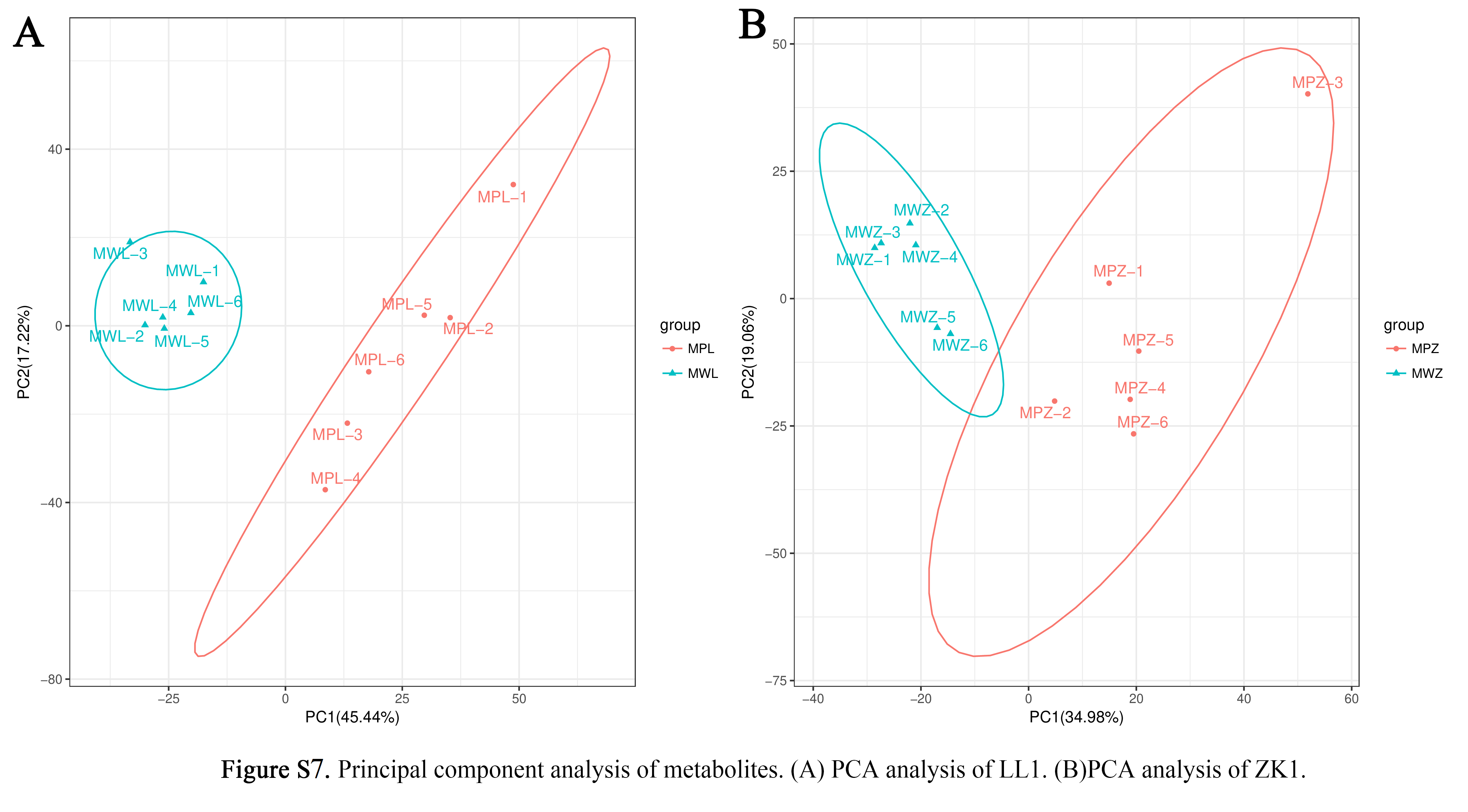

Supplement: Supplementary file 1 [file ijms-25-12188-s001.zip › Figure S7 MLLandMZZ_pca.tif]

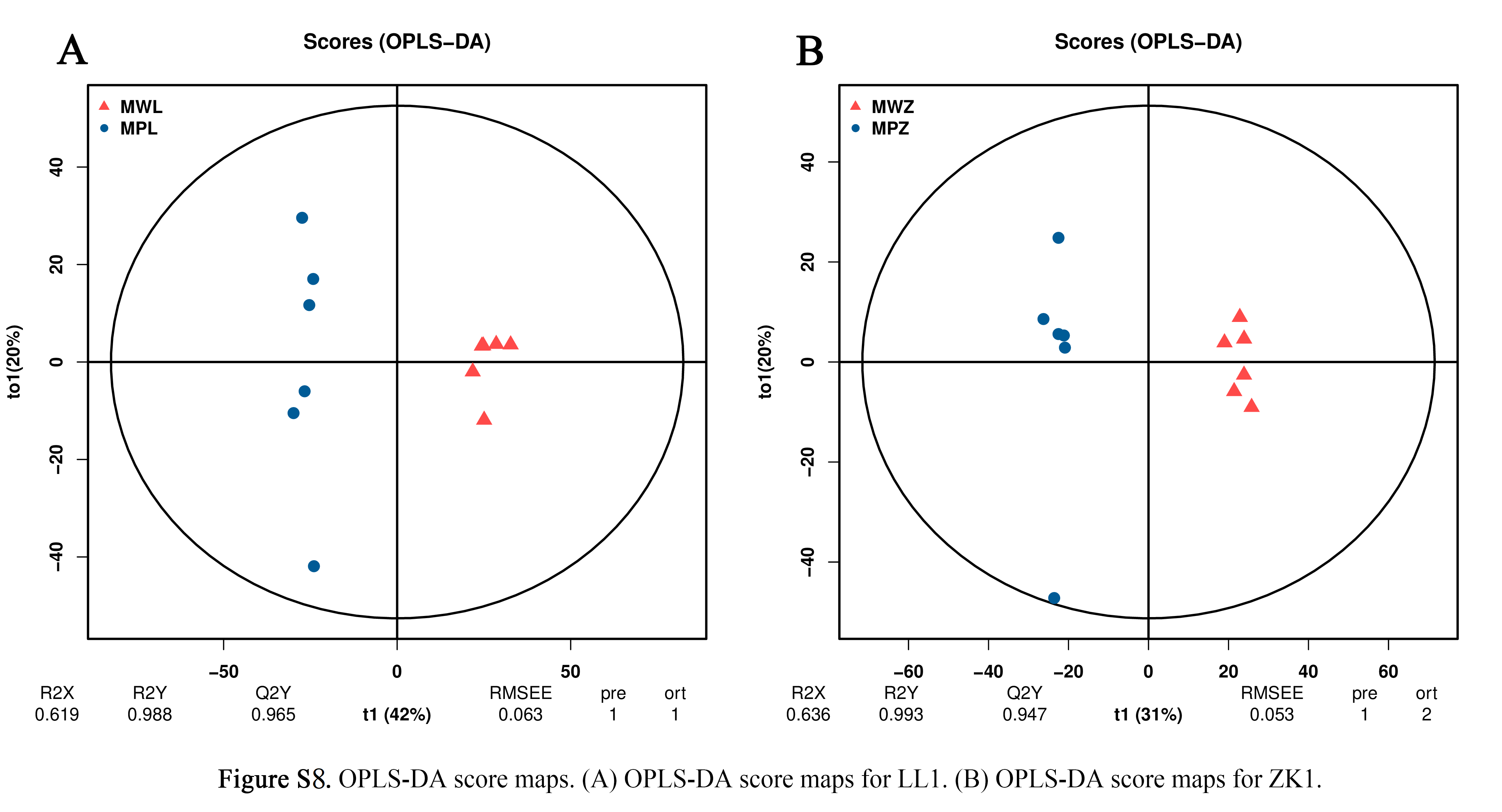

Supplement: Supplementary file 1 [file ijms-25-12188-s001.zip › Figure S8 OPLS-DA score mapsS.tif]

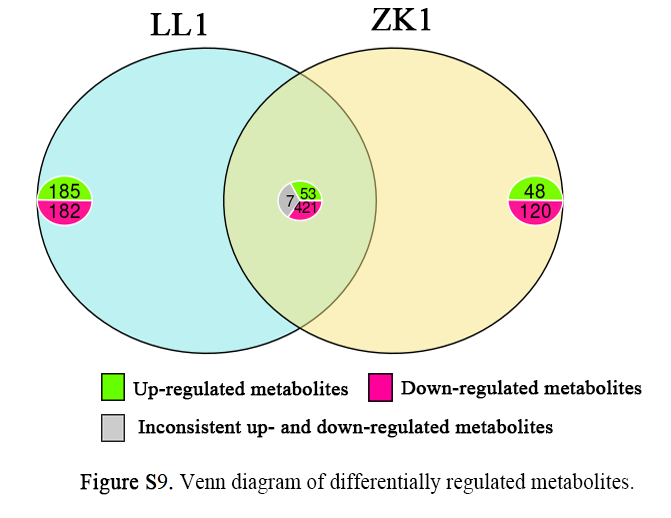

Supplement: Supplementary file 1 [file ijms-25-12188-s001.zip › Figure S9 MetaVennLvsZ.tif]
